# Spacer-Length-Dependent Nuclearity and Cuprophilic Modulation in Copper(I) Complexes with Multidentate β‑Thioketiminate Ligands

**DOI:** 10.1021/acs.inorgchem.5c04931

**Published:** 2026-01-09

**Authors:** Najeeb Ullah, Venkata Sai Sashankh Penki, Yu-Ting Chu, Amir Karim, Rahime Eshaghi Malekshah, Sodio C. N. Hsu

**Affiliations:** † Department of Medicinal and Applied Chemistry, Kaohsiung Medical University, Kaohsiung 80708, Taiwan; ‡ International PhD Program for Science, 34874 National Sun Yat-Sen University, Kaohsiung 80059, Taiwan; § Department of Medical Research, 38023 Kaohsiung Medical University Hospital, Kaohsiung 80708, Taiwan

## Abstract

This study explores the impact of alkyl spacer length in multidentate β-thioketiminate ligands (H_2_^2^ **L**-H_2_^4^ **L**, H_2_^6^ **L**, and H_2_^8^ **L**) on the nuclearity and structural properties of copper­(I) complexes. Ligands, synthesized thiation of β-ketoiminate precursors, were characterized by ^1^H, ^13^C NMR, FT-IR, UV–vis spectroscopy, and ESI-MS. Reaction with CuO^*t*^Bu led to the formation of tetranuclear ([**L**Cu_2_]_2_, C2–C4 spacers) and bis-trinuclear ([**L**Cu_2_]_3_, C6 and C8 spacers) complexes, analyzed by ^1^H, ^13^C NMR, FT-IR, UV–vis, Raman, and single-crystal X-ray diffraction. Each Cu­(I) center adopts a distorted trigonal planar geometry, coordinated by one nitrogen and two sulfur atoms through chelating and bridging modes. Shorter spacers favor tetranuclear clusters with stronger cuprophilic interactions (Cu···Cu: 2.58–3.43 Å), while longer spacers promote bis-trinuclear clusters, including a previously unreported triple-helicate with asymmetric Cu···Cu distances (2.76–2.87 Å). Importantly, coordination of 2,4,6-trimethylphenyl isocyanide induces fragmentation of these clusters into dinuclear [**L**(Cu-CNR)_2_] complexes, as confirmed by single-crystal X-ray diffraction and FT-IR (νC≡N blue shift), revealing a spacer-dependent susceptibility to isocyanide-driven S–Cu bridge cleavage. These findings demonstrate spacer length as a key modulator of Cu­(I) cluster nuclearity and metal–metal interactions, advancing bioinspired metallosupramolecular design.

## Graphical abstract

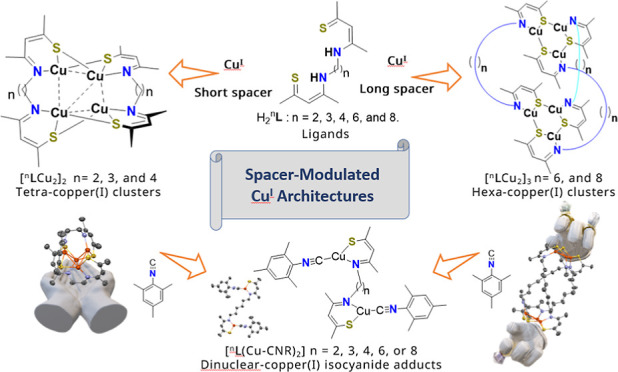

## Introduction

β-Thioketiminate ligands, distinguished by their soft sulfur and hard nitrogen donor atoms, offer flexible coordination frameworks with tunable electronic and steric properties through modifications to the ligand backbone and the *N*-aryl substituents.^1^ Ligand design,^2^ particularly the shape,^3^ size,^4^ and denticity,^5^ critically influence the nuclearity and geometry of metal-based coordination assemblies. Notably, ligands with nitrogen and sulfur donors excel at stabilizing low-valent mono-,^5,6^ and poly nuclear^1,5^ transition metal complexes. For instance, SN-type chelator ligands typically form mononuclear or sulfur-bridged multinuclear copper­(I) clusters,^1,5,7^ while higher-denticity SNN-type ligands favor discrete mononuclear species.^5,8^ Interestingly, SNS-type pincer ligands, extensively studied by Baker and co-workers, have proven effective in stabilizing diverse metal complexes, including tetranuclear copper­(I) thiolate complexes.^9,10^

Copper­(I)-thiolate chemistry has garnered significant attention due to its central role in the structural and functional behavior of cysteine-rich Cu­(I)-binding biomolecules, including copper metallothioneins,^11,12^ chaperones,^13^ and phytochelatins.^14^ The strong affinity of Cu­(I) for sulfur donor ligands facilitates the formation of robust Cu–S linkages, often giving rise to structurally diverse architectures.^1,5–7,10^ Moreover, the pronounced tendency of Cu­(I) to participate in cuprophilic (Cu···Cu) interactions renders it particularly favorable for the construction of polynuclear assemblies, including tetra- and hexanuclear clusters.^1,15–20^ The Cu­(I)···Cu­(I) distances are strongly influenced by both the ring size and the steric demands of the coordinating ligands, with increased steric bulk often promoting enhanced cuprophilic interactions.^1,21^ However, the role of alkyl spacer length in tuning these interactions remains underexplored.

Polynuclear metallohelicates have attracted considerable interest in recent years, emerging as a prominent area within the rapidly developing field of metallosupramolecular chemistry.^22^ In 1987, Lehn introduced the term metallohelices to describe a bimetallic, helical, double-stranded complex comprising two oligobipyridine ligands wrapped around two tetrahedral Cu­(I) ions.^23^ Since then, double-helical homo- and heterometallohelices have become widespread in supramolecular chemistry, particularly with metal ions such as Zn^2+^, Cu^+^, Cu^2+^, Ni^2+^, Rh^2+^, Pd^2+^, and Ru^2+^.^24–27^ Additionally, triple-helical homo- and heterometallohelices have been reported using Fe^2+^, Fe^3+^, Co^2+^, Zn^2+^, Cu^2+^, Ni^2+^, Ga^2+^, and Ir^2+^.^28–32^ Notably, triple-helical copper­(I) complexes remain unreported, presenting a significant research gap.

In this study, we designed and synthesized SNNS-type β-thioketiminate ligands with flexible alkyl spacers, which impart enhanced conformational adaptability and influence the coordination behavior of Cu­(I) thiolate complexes. Through systematic comparison with SN-, SNN-, and _R_SNS_H_-type ligands (Chart 1), we elucidate key coordination features. This study systematically examines how the *N*-alkyl spacer length influences the nuclearity of Cu­(I) thiolate clusters, with shorter spacers favoring tetranuclear and longer spacers promoting bis-trinuclear clusters, alongside modulating cuprophilic interactions. This study further investigates the reaction of Cu­(I) clusters with isocyanides, which induces fragmentation into dimeric Cu­(I) complexes and disrupts the S–Cu bridging interactions. While similar cluster cleavage has been reported for *N*-aryl-substituted Cu­(I) complexes upon treatment with isocyanides,^5^ which represents the study of isocyanide-induced fragmentation in *N*-alkyl-substituted Cu­(I) clusters.

**Chart 1:**
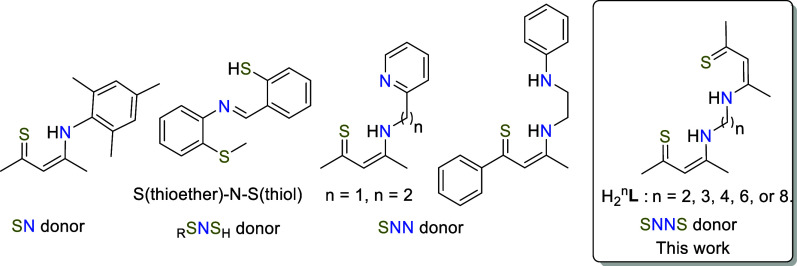
Overview of SN Donor Ligand Systems and Their Structural Motifs

## Results and Discussions

### Synthesis and Characterizations of β-Thioketiminate Ligands

A series of SNNS-type β-thioketiminate ligands (H_2_^2^ **L**, H_2_^3^ **L**, H_2_^4^ **L**, H_2_^6^ **L**, and H_2_^8^ **L**) with varying *N*-alkyl spacer lengths was synthesized through thiation of *N*-alkyl β-ketoiminate (ONNO) precursors^33–35^ using Lawesson’s reagent,^1,5,8,35^ achieving in good yields, as illustrated in Scheme 1. The diprotic ligands H_2_^2^ **L**, H_2_^3^ **L**, H_2_^4^ **L**, and H_2_^6^ **L** align with previously reported syntheses,^34,35^ while the H_2_^8^ **L** were prepared using analogous methods. The thiation reactions proceeded under mild conditions and typically reached completion within one hour. The crude products were purified by column chromatography using a DCM/hexane (3:1) eluent system to afford the ligands in good yields.

**Scheme 1:**
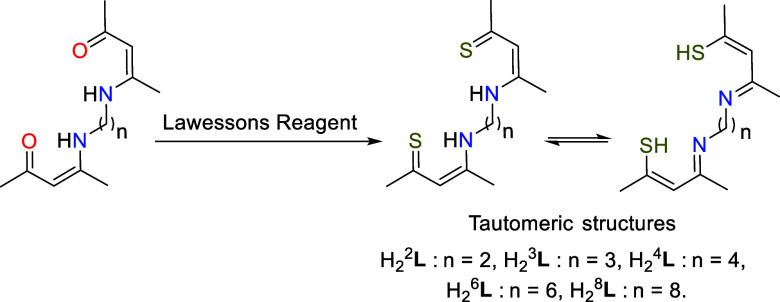
Synthetic Route to β-Thioketiminate Ligands via Thiation of *N*-Alkyl β-Ketoiminate Precursors, Including Possible Tautomeric Forms

Their purity and structural integrity were verified by comprehensive characterization, including ^1^H/^13^C NMR, FT-IR, UV–vis, and ESI-MS. The ^1^H NMR spectra of the β-ketoiminate (Nac-Ac) precursors exhibited the characteristic –NH resonance at: δ 10.83–10.87 ppm. In the corresponding β-thioketiminate (Nac-Sac) ligands (H_2_^2^ **L**-H_2_^4^ **L**, H_2_^6^ **L**, and H_2_^8^ **L**), this signal shifted markedly downfield to: δ 14.05–14.24 ppm, consistent with sulfur incorporation (Figures S1–S5). Similarly, the backbone –CH proton shifted from: δ 4.86–4.96 ppm in the Nac-Ac precursors to: δ 6.09–6.13 ppm in the Nac-Sac ligands, in agreement with values reported for related Sac-Nac systems.^1,5,35^ The ^13^C NMR spectra showed distinct deshielded C=S resonances at 195.67 ppm (H_2_^2^ **L**), 203.72 ppm (H_2_^3^ **L**), 203.74 ppm (H_2_^4^ **L**), 202.61 ppm (H_2_^6^ **L**), and 203.04 ppm (H_2_^8^ **L**), aligning with literature values for (Nac-Sac) systems (Figures S6–S10).^1,5,35,36^

### Synthesis and Spectroscopic Characterization of β-Thioketiminato Copper­(I) Complexes

The synthesis of the copper­(I) complexes employed CuO^*t*^Bu as a highly soluble and effective Cu­(I) precursor, whose high reactivity toward protic proligands facilitates the rapid formation of the corresponding Cu­(I) complexes.^37–39^ Treatment of the β-thioketiminato ligands H_2_^2^ **L**-H_2_^4^ **L**, H_2_^6^ **L**, and H_2_^8^ **L**, with CuO^*t*^Bu afforded the corresponding tetranuclear ([^2^ **L**Cu_2_]_2_ (**1**), [^3^ **L**Cu_2_]_2_ (**2**), [^4^ **L**Cu_2_]_2_ (**3**)), and hexanuclear ([^6^ **L**Cu_2_]_3_ (**4**) and [^8^ **L**Cu_2_]_3_ (**5**)) copper­(I) complexes, as depicted in Scheme 2. Subsequent reaction of these clusters with 2,4,6-trimethylphenyl isocyanide, or direct treatment of the ligands with CuO^*t*^Bu and isocyanide, produced binuclear isocyanide adducts [^2^ **L**(Cu-CNR)_2_] (**6**), [^3^ **L**(Cu-CNR)_2_] (**7**), [^4^ **L**(Cu-CNR)_2_] (**8**), [^6^ **L**(Cu-CNR)_2_] (**9**), and [^8^ **L**(Cu-CNR)_2_] (**10**). All complexes were characterized by ^1^H/^13^C NMR, UV–vis, Raman, and single-crystal X-ray diffraction, with the complexes **6**–**10** further analyzed by FT-IR to confirm the C≡N binding mode.^1,40^

**Scheme 2:**
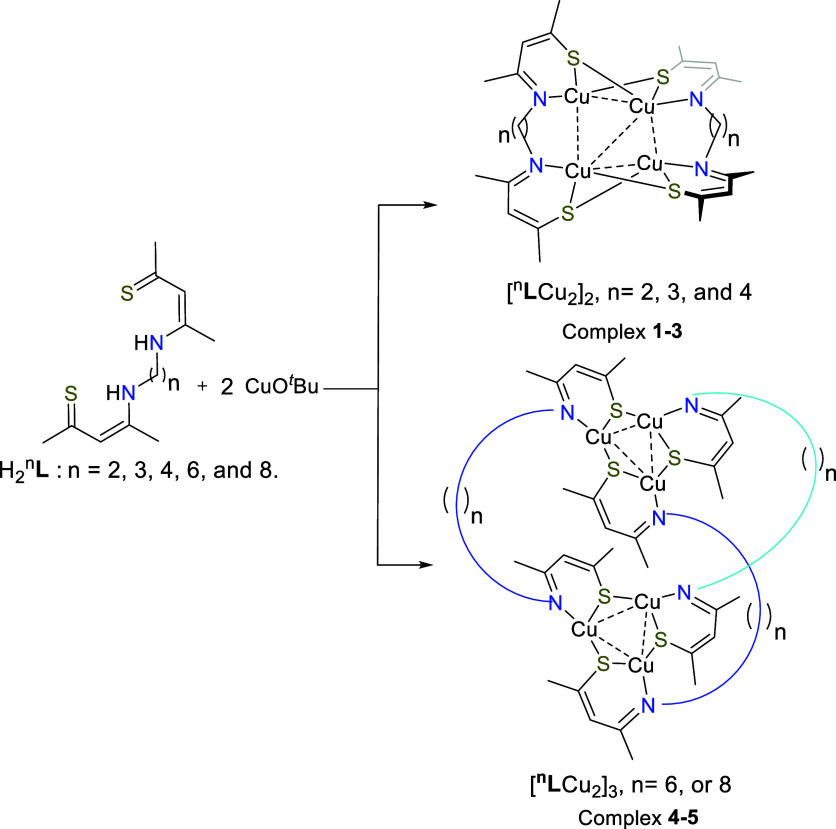
Synthesis of β-Thioketiminato copper­(I) Complexes Derived from Ligands H_2_^2^ **L**-H_2_^4^ **L**, H_2_^6^ **L**, and H_2_^8^ **L**

^1^H NMR spectra of complexes **1**–**5** demonstrated the absence of the –NH proton signal (14.05–14.24 ppm) present in the uncoordinated ligands, accompanied by a significant shift in the backbone-CH proton signals, confirming successful complexation (Figures S11–S15).^1^ Complementary FT-IR data further support this conclusion: the uncoordinated ligand displays a distinct N–H stretching vibration at 3123 cm^–1^, which disappears entirely in the corresponding Cu­(I) complex Figure S40). The combined loss of the –NH proton resonance in the NMR spectra and the disappearance of the N–H stretching band in the IR spectra provide consistent and compelling evidence for ligand deprotonation and coordination to the Cu­(I) centers, supporting the proposed binding mode in these complexes. In the ^13^C NMR spectra, the C=S resonances of H_2_^2^ **L**-H_2_^4^ **L**, H_2_^6^ **L**, and H_2_^8^ **L** (195.67–203.74 ppm) shift upfield to 167.78–165.84 ppm upon coordination, indicative of electronic changes induced by Cu­(I) binding (Figures S16–S20). Similarly, the C=N carbons signals shifted from 163.04 to 166.36 ppm in the uncoordinated ligands to 151.74–159.24 ppm in the complexes, while backbone carbons moved downfield from 96.23 to 113.49 ppm to 122.80–123.62 ppm. These chemical shift variations in both ^1^H and ^13^C NMR spectra provide compelling evidence for the formation of tetra- and hexanuclear Cu­(I) complexes, consistent with the expected coordination modes of the dianionic SNNS-type ligands, which favor polynuclear assemblies.

### Molecular Structures of β-Thioketiminate Copper­(I) Complexes

Single crystals of tetra- and bis-trinuclear copper­(I) β-thioketiminate complexes suitable for X-ray diffraction were obtained from the concentrated solution of THF (complex **1**) and MeCN (complexes **3–5**) solutions at −20 °C under a nitrogen atmosphere. In all structures, each Cu­(I) center exhibits a trigonal planar geometry, coordinated by two sulfur and one nitrogen donor atoms from the SNNS-type ligands, which engage in both intramolecular chelation and intermolecular bridging. This dual coordination mode results in multinuclear Cu­(I) assemblies with distinct nuclearities and topologies strongly influenced by the length of the *N*-alkyl spacer.

The tetranuclear complexes [^2^ **L**Cu_2_]_2_ (**1**) and [^4^ **L**Cu_2_]_2_ (**3**) exhibit a butterfly like (Cu_2_S_2_N_2_)_2_ core, consistent with reported Cu­(I) motifs (Figure 1).^7,16,19^ For [^2^ **L**Cu_2_]_2_, the two-carbon spacer enforces a compact and rigid core with the Cu···Cu distances of 2.586(4) Å (Cu_1_–Cu_3_), a central spine distance (Cu_1_–Cu_2_) of 2.854(4) Å, and a wing-tip separation (Cu_3_–Cu_4_) of 2.962(5) Å, giving an overall range of 2.586–2.962 Å and an average distance of 2.750 Å (Figure 1a,c). These short Cu···Cu separations indicate strong cuprophilic interactions, comparable to the reported averages of 2.828 Å for similar butterfly type Cu­(I) clusters.^16,19^ In contrast, the four-carbon spacer in [^4^ **L**Cu_2_]_2_ leads to a more expanded tetranuclear framework with Cu···Cu distances of 2.704(8) Å (Cu_1_–Cu_3_), a central spine distance 3.368 Å (Cu_1_–Cu_2_), and a wing-tip separation 3.435 Å (Cu_3_–Cu_4_), giving an overall range of 2.704–3.435 Å and an average distance of 2.982 Å (Figure 1b,d). This elongation reflects reduced cuprophilic interaction strength and increased core flexibility compared to [^2^ **L**Cu_2_]_2_ and reported structures.^16^ Rather than crystal structures, (Figure 1e,f) present the ChemDraw representations of the [^2^ **L**Cu_2_]_2_ and [^4^ **L**Cu_2_]_2_ complexes, providing a clearer visualization of the ligand-coordination modes for improved understanding.

**Figure 1:**
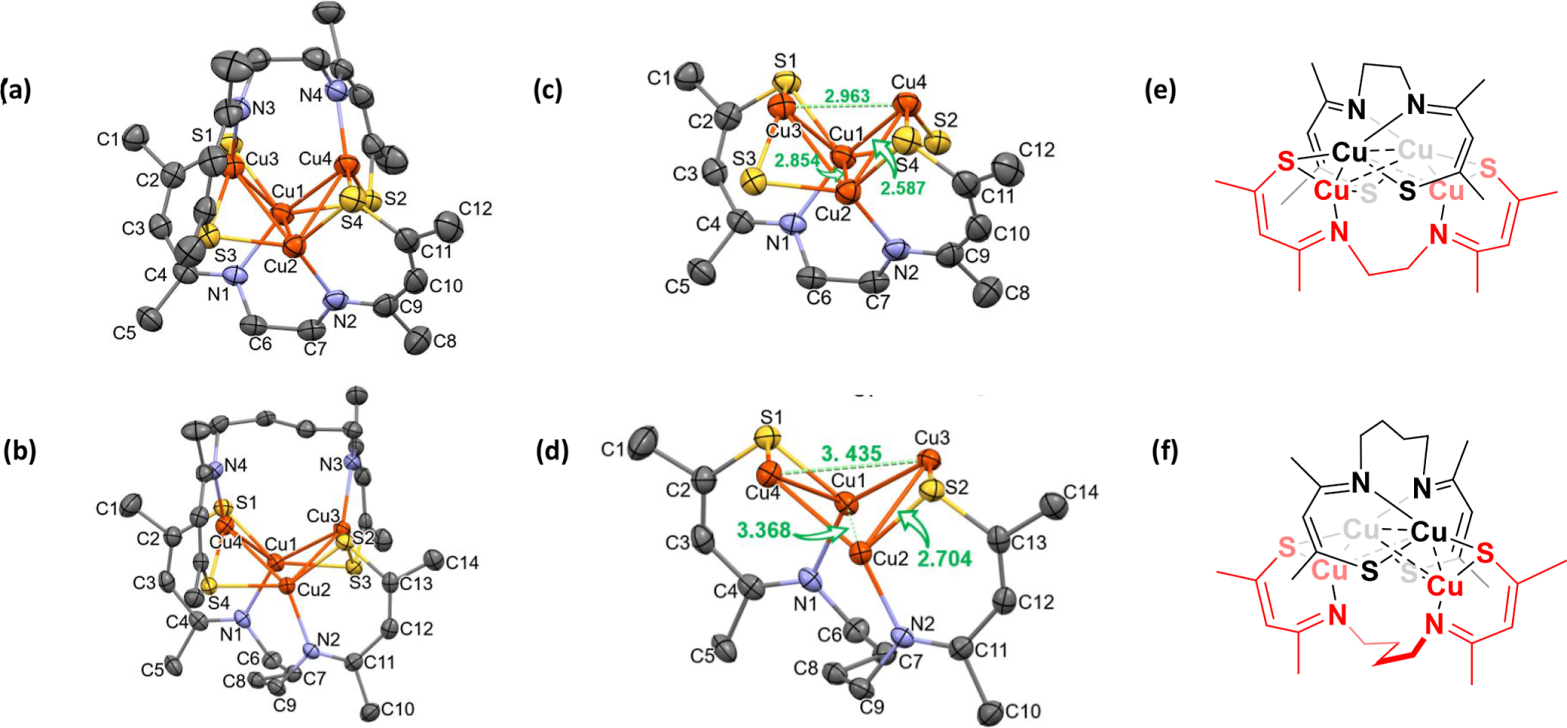
(a,b) ORTEP representations of the tetranuclear Cu­(I) complexes **1** and **3**, respectively, drawn at 20% probability ellipsoids. (c,d) Simplified views of the Cu_4_ cores in complexes **1** and **3**, emphasizing principal intracluster interactions. (e,f) ChemDraw representations of the complexes **1** and **3**, illustrating the ligand frameworks. Hydrogen atoms are omitted for clarity.

Interestingly, the extension of the spacer to six and eight carbons induces a structural transition to bis-trinuclear clusters assemblies, [^6^ **L**Cu_2_]_3_ (**4**) and [^8^ **L**Cu_2_]_3_ (**5**), adopting a (Cu_3_S_3_N_3_)_2_ architecture with three ligands bridging six Cu­(I) centers (Figure 2a,b).^41^ In [^6^ **L**Cu_2_]_3_, ligands symmetrically bridge two Cu_3_ triangular subunits, forming highly symmetric dual Cu_3_ cores with uniform Cu···Cu separations of 2.820(2) Å (Figure 2c), closely matching the literature average of 2.820 Å for hexanuclear Cu­(I) complexes.^42^ Conversely, [^8^ **L**Cu_2_]_3_ exhibits two distorted bis-trimeric cores due to the increased flexibility of the eight-carbon spacer. One ligand (N_1_–S_1_) coordinates symmetrically to Cu_1_, while the other two (S_2_–N_2_ and S_3_–N_3_) adopt twisted, asymmetric modes, bridging Cu_3_ and Cu_2_ asymmetrically (Figure 2d). This leads to a previously unreported triple-helicate structure with nonequivalent Cu···Cu distances of 2.765(9) Å (Cu_2_–Cu_3_), 2.787(10) Å (Cu_3_–Cu_1_), and 2.872(9) Å (Cu_1_–Cu_2_), giving an overall range of 2.765–2.872 Å and an average distance of 2.807 Å, indicating strong cuprophilic interactions compared to prior hexanuclear Cu­(I) analogues.^43^ Instead of crystal structures, (Figure 2e,f) present ChemDraw representations of the [^6^ **L**Cu_2_]_3_ and [^8^ **L**Cu_2_]_3_ complexes, offering a clearer visualization of the ligand coordination modes and facilitating a better understanding of their structural features. These findings demonstrate that spacer length critically governs the nuclearity, topology, and symmetry, transitioning from compact tetranuclear to symmetric or distorted bis-trinuclear architectures. The key Cu···Cu, Cu–S, and Cu–N bond distances, as well as the principal bond angles around the copper centers, are summarized in Table 1, with comprehensive data in Table S1. These structural insights highlight the profound influence of ligand design on the formation and properties of Cu­(I) clusters, offering a robust platform for tailoring metallosupramolecular assemblies.

**Figure 2:**
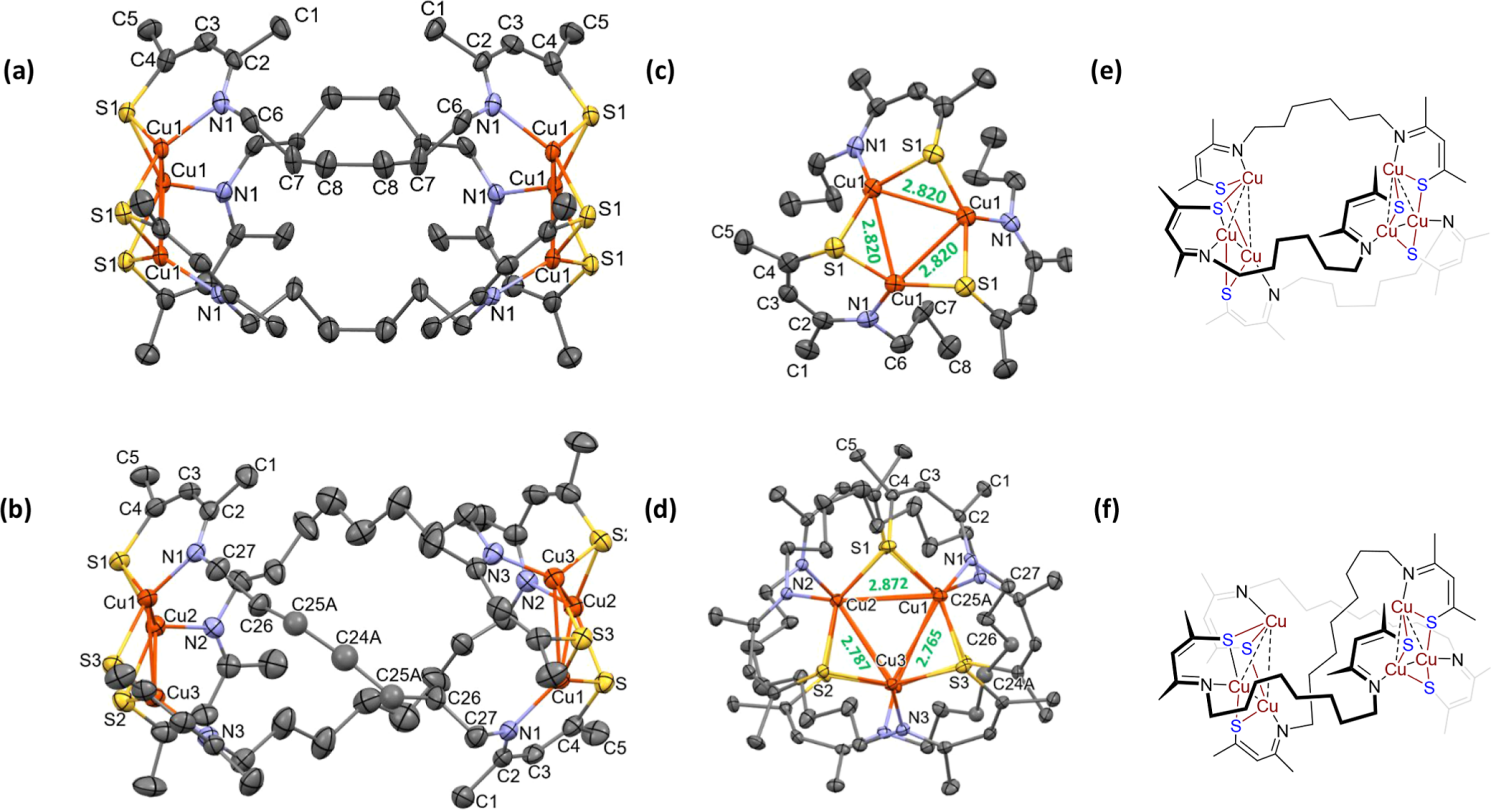
(a,b) ORTEP diagrams (side views) of the bis-trinuclear Cu­(I) complexes **4** and **5**, respectively, drawn with 20% probability ellipsoids. (c,d) Enlarged top-view representations of the Cu_6_ cores, highlighting the highly symmetric core in complex **4** and the distorted, asymmetric Cu_6_ framework in complex **5**. (e,f) ChemDraw illustrations of complexes **4** and **5**, showing the ligand frameworks. All hydrogen atoms are omitted for clarity.

**Table 1:** Selected Average Bond Distances (Å) and Bond Angles (°) for Complexes [^2^ **L**Cu_2_]_2_, [^4^ **L**Cu_2_]_2_, [^6^ **L**Cu_2_]_3_, and [^8^ **L**Cu_2_]_3_

	[^2^ **L**Cu_2_]_2_	[^4^ **L**Cu_2_]_2_	[^6^ **L**Cu_2_]_3_	[^8^ **L**Cu_2_]_3_
Cu–Cu_ave_	2.750 ± 0.163	2.982 ± 0.278	2.820 ± 0.002	2.807 ± 0.043
Cu–S_(chelated)_	2.231 ± 0.026	2.186 ± 0.005	2.215 ± 0.003	2.199 ± 0.005
Cu–S_(bridged)_	2.263 ± 0.004	2.260 ± 0.010	2.213 ± 0.003	2.222 ± 0.003
Cu–N	1.977 ± 0.009	2.012 ± 0.003	1.976 ± 0.008	1.963 ± 0.002
S_(chelate)_CuS_(bridge)_	126.65 ± 9.00	137.70 ± 1.23	124.30 ± 0.14	123.77 ± 0.74
N–Cu–S_(bridge)_	126.40 ± 9.24	111.82 ± 1.78	131.00 ± 0.00	128.54 ± 0.44
N–Cu–S_(chelate)_	105.54 ± 0.15	104.78 ± 0.35	103.90 ± 0.00	105.77 ± 0.37

### Raman Spectroscopic Analysis of β-Thioketiminato Cu­(I) Complexes

Raman spectroscopy was employed to investigate intramolecular cuprophilic interactions in tetra- and bis-trinuclear β-thioketiminato Cu­(I) complexes (**1**–**5**) and their isocyanide adducts (**6**–**10**), focusing on the characteristic ν­(Cu–Cu) stretching vibrations, which are challenging to assign unambiguously due to the predominance of studies on ligand-unsupported intermolecular interactions.^27,44–47^ In contrast, relatively few investigations have addressed ligand-supported intramolecular cuprophilic interactions.^1,7^ Solid-state Raman spectra, collected using a 633 nm diode laser, revealed well-resolved ν­(Cu–Cu) bands for complexes **1**–**5** at 204.29 cm^–1^ (**1**, average Cu···Cu distance = 2.750 Å), 207.27 cm^–1^ (**2**), 213.22 cm^–1^ (**3**, 2.982 Å), 207.27 cm^–1^ (**4**, 2.820 Å), and 206.99 cm^–1^ (**5**, 2. 807 Å) (Figures S32 and S33, red spectra). The observed ν­(Cu–Cu) frequencies correlate closely with the crystallographically determined Cu···Cu distances, reinforcing presence of intramolecular cuprophilic interactions and supporting the structural assignments. Upon coordination with 2,4,6-trimethylphenyl isocyanide, the ν­(Cu–Cu) bands in complexes **6**–**10** were absent (Figures S32 and S33, blue spectra), indicating disruption of the Cu···Cu interactions. This observation is consistent with previous reports of isocyanide-induced fragmentation in Cu­(I) clusters.^1^ The ν­(Cu–S) and ν­(Cu–N) vibrational modes were assigned by comparing parent clusters with their adducts and referencing literature on Cu­(I) complexes with sulfur or nitrogen donors.^1,18,45,48^ The ν­(Cu–S) bands appeared in the 250–300 cm^–1^ region, while ν­(Cu–N) modes were observed between 300 and 400 cm^–1^, aligning with expected values for these coordination environments. These Raman data underscore the sensitivity of vibrational spectroscopy in probing cuprophilic interactions and highlight the role of ligand design in modulating the structural and electronic properties of Cu­(I) complexes, offering insights for bioinspired metallosupramolecular applications.

### Isocyanide-Induced Nuclearity Reduction in β-Thioketiminato Cu­(I) Complexes

The coordination behavior of d^10^ Cu­(I) centers is strongly influenced by isocyanides, which act as potent σ-donors with minimal π-acceptor character.^1,5,40^ This electronic profile favors σ-donation over π-backbonding, typically leading to a blue shift in the isocyanide stretching frequency (νC≡N) upon coordination. To probe the electron-donating properties of the *N*-alkyl-substituted β-thioketiminato Cu­(I) fragments, tetra- or bis-trinuclear copper­(I) clusters (**1**–**5**) were reacted with 2,4,6-trimethylphenyl isocyanide, affording binuclear ligand-dicopper­(I)-isocyanide (1:2:2) complexes [^2^ **L**(Cu**-**CNR)_2_] (**6**), [^3^ **L**(Cu**-**CNR)_2_] (**7**), [^4^ **L**(Cu-CNR)_2_] (**8**), [^6^ **L**(Cu**-**CNR)_2_] (**9**), and [^8^ **L**(Cu-CNR)_2_] (**10**). Alternatively, direct treatment of ligands H_2_^2^ **L**-H_2_^4^ **L**, H_2_^6^ **L**, and H_2_^8^ **L** with CuO^*t*^Bu and isocyanide afforded the same adducts (Scheme 3). Monoisocyanide copper­(I) complexes have been extensively investigated in the literature,^1,5,37,38,49^ however, the dicopper­(I) diisocyanide counterparts remain relatively unexplored.^40^ The present dicopper­(I) isocyanide adducts (**6–10**) were comprehensively characterized by ^1^H/^13^C NMR spectroscopy, Raman and FT-IR analyses, as well as single-crystal X-ray crystallography.

**Scheme 3:**
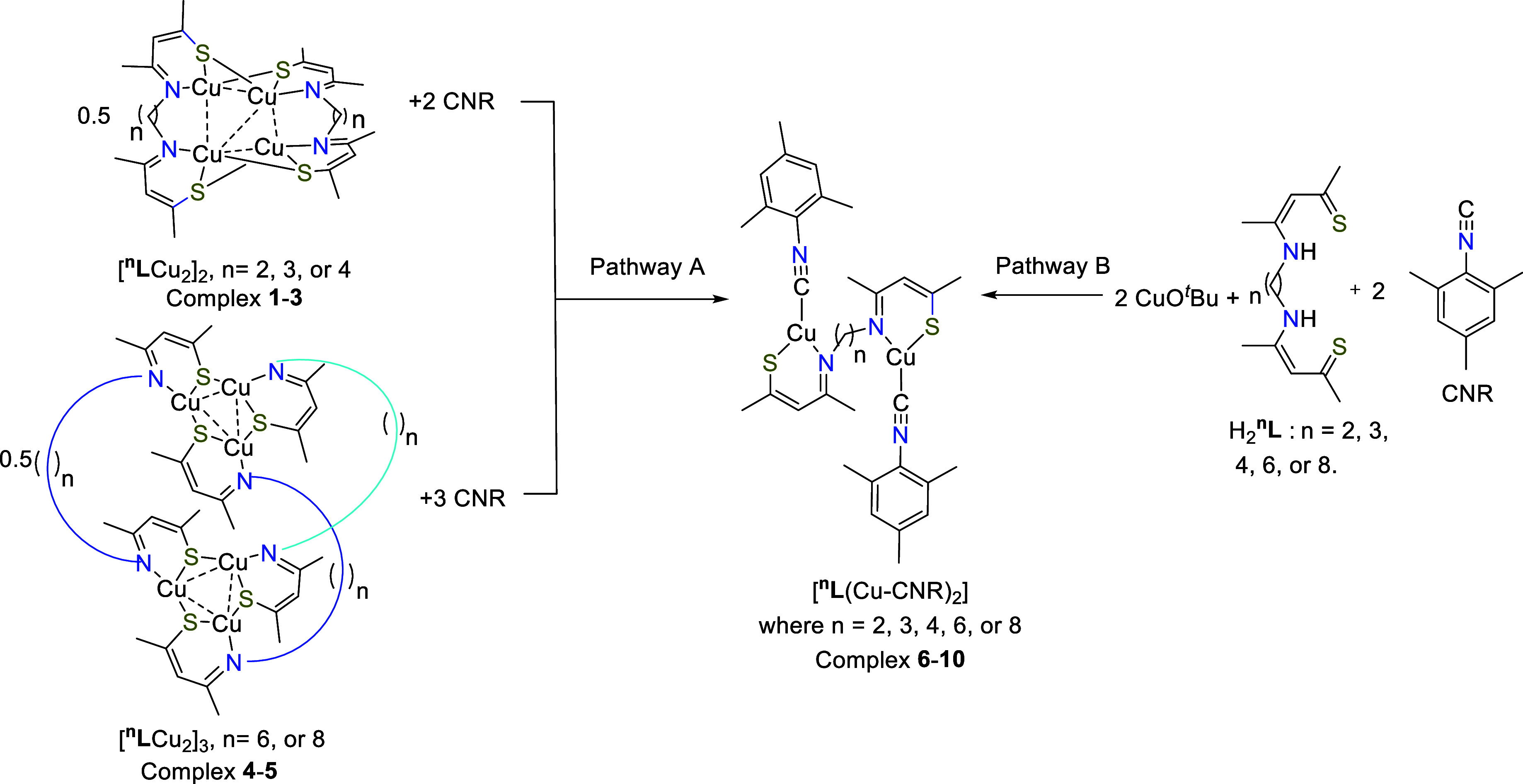
Two Synthetic Pathways for the Preparation of Binuclear copper­(I) Isocyanide Adducts [^**n**^ **L**(Cu-CNR)_**2**_] from β -Thioketiminate Ligands and Cu­(I) Clusters

In the ^1^H NMR spectra of complexes **6**–**10**, the aromatic *meta* protons appear at: δ 6.87–6.90 ppm, the *ortho*-methyl protons at: δ 2.30–2.33 ppm, and the *para-*methyl protons at: δ 2.04–2.06 ppm. These characteristic resonances provide clear evidence for successful isocyanide coordination in the binuclear copper complexes, as illustrated in Figures S21–S25.

The solution-state behavior of the β-thioketoiminato ligand framework and its copper­(I) complexes was probed by ^1^H NMR spectroscopy. Figure 3 presents a representative comparison of the spectra of the free ligand H_2_^2^ **L**, the tetranuclear cluster [^2^ **L**Cu_2_]_2_, and the isocyanide-derived dinuclear adduct [^2^ **L**(Cu-CNAr)_2_], with proton assignments indicated on the accompanying ChemDraw structures. In the uncoordinated ligand H_2_^2^ **L**, the NH–CH_2_–CH_2_–NH fragment displays a pseudotriplet.^50^ However, it is not immediately evident whether this pattern originates from the coupling between the NH and adjacent CH_2_ groups or from the mutual coupling within the CH_2_–CH_2_ ethylene segment. Upon formation of the tetranuclear cluster [^2^ **L**Cu_2_]_2_, this ethylene spacer is constrained within a cyclic chelate environment imposed by the Cu_4_S_4_ core. As a result, the methylene protons become diastereotopic, manifesting as two well-resolved doublets instead of the original pseudotriplet. This pronounced magnetic nonequivalence directly reflects the rigid, unsymmetrical coordination geometry experienced by the ligand in the clusters mentioned in Figure 1a. Addition of two equivalents of 2,4,6-trimethylphenyl isocyanide triggers complete disassembly of the tetranuclear framework into the dinuclear complex [^2^ **L**(Cu-CNAr)_2_]. Remarkably, the N–CH_2_–CH_2_–N resonance collapses back to a sharp singlet, indicating restoration of effective time-averaged symmetry about the ethylene bridge. This dramatic simplification of the spectrum confirms that isocyanide coordination releases the conformational constraints imposed by the cluster core, allowing rapid conformational averaging of the flexible spacer on the NMR time scale. The ^13^C NMR spectra further support isocyanide coordination, with characteristic signals for the isocyanide carbon (C≡N) at 165.71–167.22 ppm and the adjacent C≡N–C carbon at 139.68–139.84 ppm. Aromatic carbons corresponding to *ortho*, *meta*, and *para* positions appear at: δ 134.82–134.97, 128.20–128.91, and 120.12–124.11 ppm, respectively, confirming the formation of the isocyanide adducts (Figures S26–S30). These diagnostic spectral changes underscore the exceptional structural adaptability of the bis­(β-thioketoiminato) platform and provide clear solution-phase evidence of the dynamic interplay between ligand conformation, cluster nuclearity, and external ligation at the copper­(I) centers.

**Figure 3:**
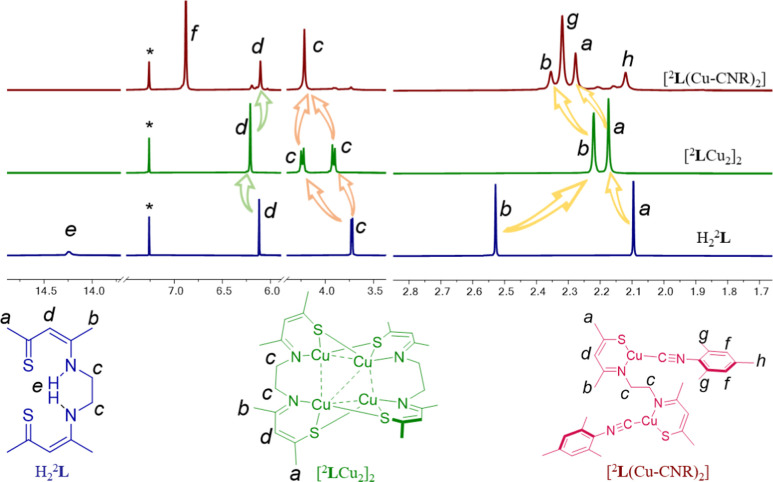
Overlayed ^1^H NMR spectra of H_2_^2^ **L** (bottom), tetranuclear [^2^ **L**Cu_2_]_2_ (middle), and the binuclear isocyanide adduct [^2^ **L**(Cu-CNR)_2_] (top), highlighting coordination-induced shifts. Asterisks (*) denote residual CDCl_3_ signals.

FT-IR analysis revealed a blue shift in the νC≡N stretching frequency from 2119 cm^–1^ (uncoordinated isocyanide) to 2133–2135 cm^–1^ upon coordination to Cu­(I), as shown in Figure S39. This shift reflects reduced π-backbonding from the copper center to the isocyanide ligand, consistent with the expected behavior for d^10^ Cu­(I) systems.^40,51^ Furthermore, the *N*-alkyl-substituted β-thioketiminato ligands displayed weaker electron-donating abilities compared to their *N*-aryl-substituted counterparts,^1,5^ suggesting that the nature of the *N-*alkyl substituent exerts only a minor influence on the overall donor strength of the ligand framework discussed in this study. These findings align well with previous reports on copper­(I)-isocyanide systems and reinforce the role of ligand design in modulating metal–ligand electronic interactions.^1,5,40^

### Molecular Structures of Binuclear β-Thioketiminato Cu­(I) Isocyanide Adducts

Single crystals of the binuclear copper­(I) isocyanide adduct [^6^ **L**(Cu-CNR)_2_] and [^8^ **L**(Cu-CNR)_2_] were obtained from slow crystallization in toluene at −20 °C under an inert atmosphere. X-ray crystallographic analysis revealed a distinctive *Z* **-**shaped topology, driven by a flexible SNNS-type bidentate ligand with six-carbon (^6^ **L**) or eight-carbon (^8^ **L**) aliphatic spacers (Figure 4). Each copper­(I) center adopts a trigonal planar geometry, coordinated by one nitrogen and one sulfur atom from the β-thioketiminate ligand and one carbon atom from a 2,4,6-trimethylphenyl isocyanide (C≡NR) ligand, establishing a 1:2:2 (ligand/metal/isocyanide) stoichiometry stabilized by robust metal–ligand interactions.^1,5,40^

**Figure 4:**
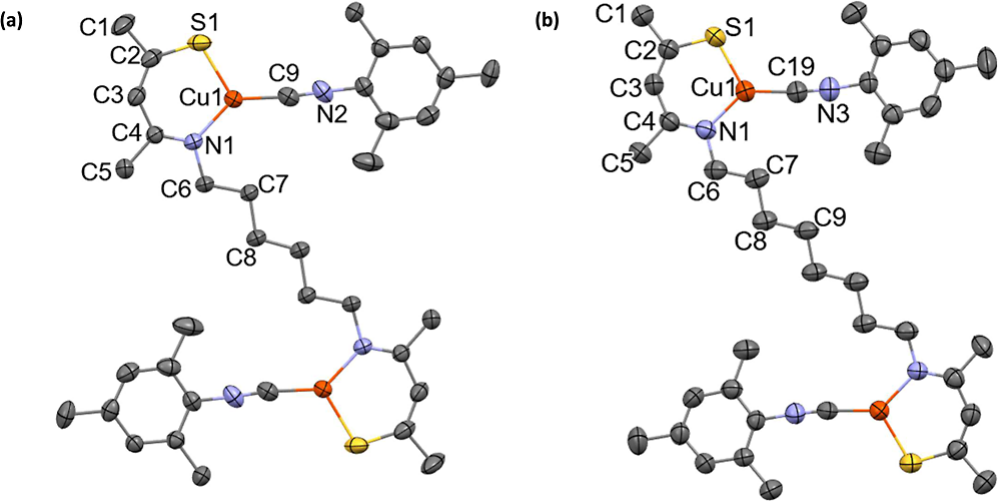
ORTEP representations of the isocyanide adducts (a) [^6^ **L**(Cu-CNR)_2_] and (b) [^8^ **L**(Cu-CNR)_2_], shown with 25% probability ellipsoids. Hydrogen atoms have been omitted for clarity.

The key average bond lengths for [^6^ **L**(Cu-CNR)_2_] include Cu–N, 1.957(14) Å, Cu–S, 2.203(5) Å, and Cu–C, 1.850(19) Å, while those for [^8^ **L**(Cu-CNR)_2_] are Cu–N, 1.960(2) Å, Cu–S, 2.192(8) Å, and Cu–C, 1.847(3) Å, consistent with strong three-coordinate Cu­(I) bonding (Table S3).^1,40^ The slightly elongated Cu–S bonds in [^8^ **L**(Cu-CNR)_2_] reflects increased flexibility and reduced steric strain due to the longer eight-carbon spacer. The trans orientation of the isocyanide ligands minimizes steric hindrance, promoting effective intermolecular packing. This modular ligand design enables precise control over the Cu­(I) coordination environment, with the extended spacer in [^8^ **L**(Cu-CNR)_2_] enhancing conformational adaptability compared to [^6^ **L**(Cu-CNR)_2_]. These structural insights underscore the critical role of alkyl spacer length in tuning the geometric and supramolecular properties of binuclear Cu­(I) complexes, offering flexible platform for designing metallosupramolecular architectures with tailored characteristics.

## Conclusions

This work addresses a significant gap in copper­(I) cluster chemistry by elucidating the role of *N*-alkyl spacer length in β-thioketiminato ligands in modulating cluster nuclearity and cuprophilic interactions. Through systematic variation of spacer lengths, we demonstrate that shorter spacers (C_2_–C_4_) promote compact tetranuclear butterfly like structures ([^2^ **L**Cu_2_]_2_-[^4^ **L**Cu_2_]_2_) with strong Cu···Cu interactions (2.586–2.962 Å), as confirmed by X-ray diffraction. In contrast, longer spacers (C_6_–C_8_) drive the formation of previously unreported bis-trinuclear triple-helical architectures ([^6^ **L**Cu_2_]_3_, [^8^ **L**Cu_2_]_3_), with the six-carbon spacer yielding a symmetric dual Cu_3_ core (Cu···Cu: 2.820 Å) and the eight-carbon spacer introducing flexibility, resulting in a distorted, asymmetric core (Cu···Cu: 2.765–2.872 Å). Coordination with 2,4,6-trimethylphenyl isocyanide induces fragmentation into binuclear [**L**(Cu-CNR)_2_] complexes, disrupting the S–Cu bridges and marking the earliest known example of isocyanide-induced cleavage in *N*-alkyl spacer-substituted Cu­(I) clusters. These findings establish spacer length as a critical design parameter for tailoring butterfly like and triple-helical Cu­(I) architectures, offering insights for engineering metallosupramolecular systems with tunable metal–metal interactions for bioinspired applications.

## Experimental Section

### Materials and Methods

All experimental procedures were carried out under an inert dinitrogen atmosphere employing standard Schlenk line or glovebox techniques to prevent exposure to air and moisture. Copper­(I) *tert*-butoxide (CuO^*t*^Bu) and β-thioketiminate ligands were synthesized according to reported procedures.^5^ Commercial reagents were obtained from Sigma-Aldrich, Lancaster Chemicals, or Fluka and used as received without further purification. Organic solvents were purified by standard methods: toluene and tetrahydrofuran (THF) were dried over sodium metal, while dichloromethane (CH_2_Cl_2_) and acetonitrile (CH_3_CN) were dried over calcium hydride. All solvents were deoxygenated with dinitrogen prior to use. Fourier-transform infrared (FT-IR) spectra were recorded on a Bruker Alpha OPUS spectrophotometer using KBr pellets. UV–visible absorption spectra were obtained on an Agilent 8453 spectrophotometer. Nuclear magnetic resonance (^1^H and ^13^C NMR) spectra were recorded on a JEOL 400 MHz spectrometer, with tetramethylsilane (TMS) as the internal reference. Electrospray ionization mass spectrometry (ESI-MS) measurements were carried out on a Waters ZQ 4000 mass spectrometer. Single-crystal X-ray diffraction data were collected on a Rigaku Oxford XtaLAB ProII diffractometer equipped with Mo Kα radiation (λ = 0.71073 Å). Unit cell determination and data integration were performed using the Bruker SMART software package.^52^ Non-hydrogen atoms were refined anisotropically, while hydrogen atoms were placed in calculated positions and refined using a riding model. The Raman spectra were recorded on a micro-Raman spectrometer (MRID, Protrustech, Taiwan) equipped with a 633 nm diode laser (30 mW, NA = 0.55×, 50× objective). Spectra were collected with an integration time of 30 s and 10 accumulations. Samples were prepared as KBr pellets (compound: KBr = 3:10) under an inert atmosphere.

### Synthesis of Copper­(I) Complexes

[^2^ **L**Cu_2_]_2._ Under an inert atmosphere, a suspension of CuO^*t*^Bu (1.06 g, 7.80 mmol, 2 equiv) in hexane (5 mL) was treated dropwise with a solution of [H_2_^2^ **L**] (1.00 g, 3.90 mmol, 1 equiv) in THF (5 mL). The suspension changed color from yellow to orange, and the reaction mixture was stirred at room temperature for 1 h. Removal of the volatiles under reduced pressure afforded a reddish-orange solid, which was recrystallized from THF/hexane at −20 °C to yield bright orange crystals of [^2^ **L**Cu_2_]_2_ (2.43 g, 82%).^1^H NMR (CDCl_3_, 400 MHz, 298 K, δ): 6.22 (s, 2H, β-**H**), 4.26–3.91 (dd, 4H, *J* = 6.0 Hz, N–**CH**_**2**_), 2.23 (s, 6H, NC**CH**_**3**_), 2.19 (s, 6H, SC**CH**_**3**_). ^13^C NMR (CDCl_3_, 100 MHz, 298 K, δ): 166.88 (**C–**S), 159.24 (**C**=N), 122.88 (β-**C**), 57.37 (N–**C**H_2_), 34.52 (NC**C**H_3_) 23.72 (SC**C**H_3_). UV–vis: 297 nm (ε = 15,025 M^–1^·cm^–1^).

[^3^ **L**Cu_2_]_2._ Using the same synthetic protocol as for [^2^ **L**Cu_2_]_2_, the reaction of CuO^*t*^Bu (1.01 g, 7.40 mmol, 2 equiv) with [H_2_^3^ **L**] (1.00 g, 3.70 mmol) afforded [^3^ **L**Cu_2_]_2_ as an orange solid (2.36 g, 81% yield). ^1^H NMR (CDCl_3_, 400 MHz, 298 K, δ): 6.14 (s, 2H, β-**H**), 4.25–3.77 (q, 4H, *J* = 6.5 Hz, N–**CH**_**2**_), 2.25–2.22 (m, 4H, *J* = 6.0 Hz, NHCH_2_ **CH**_**2**_), 2.19 (s, 6H, NC**CH_3_**), 2.09 (s, 6H, SC**CH_3_**). ^13^C NMR (CDCl_3_, 100 MHz, 298 K, δ): 167.78 (**C–**S), 156.35 (**C**=N), 122.96 (β-**C**), 51.91 (N–**C**H_2_), 34.55 (NC**C**H_3_), 29.13 (SC**C**H_3_), 23.36 (NCH_2_ **C**H_2_). UV–vis: 325 nm (ε = 15,720 M^–1^·cm^–1^).

[^4^ **L**Cu_2_]_2._ Using the same synthetic protocol as for [^2^ **L**Cu_2_]_2_, the reaction of CuO^*t*^Bu (0.96 g, 7.20 mmol, 2 equiv) with [H_2_^4^ **L**] (1.00 g, 3.51 mmol) afforded as a brown-orange solid. Recrystallization from MeCN at −20 °C yielded bright brown-orange crystals of [^4^ **L**Cu_2_]_2_ (2.26 g, 79% yield). ^1^H NMR (CDCl_3_, 400 MHz, 298 K, δ): 6.09 (s, 2H, β-**H**), 3.91–3.73 (m, 4H, *J* = 12 Hz, N–**CH_2_**), 2.46–2.35 (m, 4H, *J* = 11 Hz, N**CH_2_**), 2.19 (s, 6H, NC**CH_3_**), 2.06 (s, 6H, SC**CH_3_**), 1.48–1.41 (t, 4H, *J* = 14 Hz, NCH_2_ **CH_2_**). ^13^C NMR (CDCl_3_, 100 MHz, 298 K, δ): 165.86 (**C–**S), 154.25 (**C**=N), 122.96 (β-**C**), 52.38 (N–**C**H_2_), 34.69 (NC**C**H_3_), 26.66 (SC**C**H_3_), 24.94 (NCH_2_ **C**H_2_). UV–vis: 344 nm (ε = 6392 M^–1^·cm^–1^).

[^6^ **L**Cu_2_]_3_. Using the same synthetic protocol as for [^2^ **L**Cu_2_]_2_, the reaction of CuO^*t*^Bu (0.87 g, 6.38 mmol, 2 equiv) with [H_2_^6^ **L**] (1.00 g, 3.19 mmol) afforded [^6^ **L**Cu_2_]_3_ as yellow solid. Recrystallization from MeCN at −20 °C yielded bright orange crystals of [^6^ **L**Cu_2_]_3_ (3.50 g, 83% yield). ^1^H NMR (CD_2_Cl_2_, 400 MHz, 298 K, δ): 6.11 (s, 2H, β-**H**), 3.62 (br, 2H, N–**CH_2_**), 3.25 (br, 2H, N**CH_2_**), 2.26 (s, 6H, NC**CH_3_**), 2.00 (s, 6H, SC**CH_3_**), 1.26 (br, 2H, NCH_2_ **CH_2_**), 1.31 (br, 2H, NCH_2_ **CH_2_**), 1.14 (br, 2H, N­(CH_2_)_2_ **CH_2_**), 1.04 (br, 2H, N­(CH_2_)_2_ **CH_2_**).^13^C NMR (CD_2_Cl_2_, 400 MHz, 298 K, δ): 166.35 (**C–**S), 157.24 (**C**=N), 123.34 (β-**C**), 34.33 (N–**C**H_2_), 32.06 (NC**C**H_3_) 29.38 (SC**C**H_3_), 27.26 (NCH_2_ **C**H_2_), 23.19 (N­(CH_2_)_2_ **C**H_2_). UV–vis: 342 nm (ε = 14,152 M^–1^·cm^–1^).

[^8^ **L**Cu_2_]_3_. Using the same synthetic protocol as for [^2^ **L**Cu_2_]_2_, the reaction of CuO^*t*^Bu (0.80 g, 5.85 mmol, 2 equiv) with [H_2_^8^ **L**] (1.00 g, 2.93 mmol) afforded [^8^ **L**Cu_2_]_3_ as a yellow solid. Recrystallization from MeCN at −20 °C yielded bright orange crystals of [^8^ **L**Cu_2_]_3_ (3.45 g, 84% yield). ^1^H NMR (CD_2_Cl_2_, 400 MHz, 298 K, δ): 6.11 (s, 2H, β-**H**), 3.66–3.54 (m, 2H, N–**CH_2_**), 3.33–3.19 (m, 2H, N**CH_2_**), 2.26 (s, 6H, NC**CH_3_**), 1.99 (s, 6H, SC**CH_3_**), 1.43–1.34 (m, 2H, NCH_2_ **CH_2_**), 1.06–0.98 (m, 2H, NCH_2_ **CH_2_**), 1.20 (br, 4H, N­(CH_2_)_2_ **CH_2_**), 1.12 (br, 4H, N­(CH_2_)_3_ **CH_2_**). ^13^C NMR (CD_2_Cl_2_, 400 MHz, 298 K, δ): 166.38 (**C–**S), 157.28 (**C**=N), 123.30 (β-**C**), 56.15 (N–**C**H_2_), 34.33 (NC**C**H_3_) 32.04 (SC**C**H_3_), 29.37 (NCH_2_ **C**H_2_), 27.25 (N­(CH_2_)_2_ **C**H_2_), 23.18 (N­(CH_2_)_3_ **C**H_2_). UV–vis: 335 nm (ε = 8800 M^–1^·cm^–1^).

### Synthesis of Binuclear β-Thioketiminato Cu­(I) Isocyanide Adducts

Two synthetic routes were employed to prepare the binuclear β-Thioketiminato Cu­(I) isocyanide complexes **6**–**10**.

#### Pathway A

In a glovebox, a solution of the copper­(I) complexes [^2^ **L**Cu_2_]_2_, [^3^ **L**Cu_2_]_2,_ [^4^ **L**Cu_2_]_2,_ [^6^ **L**Cu_2_]_2_ or [^8^ **L**Cu_2_]_2_ (1.00 g, 1.0 equiv) in 5 mL of THF was prepared. To each solution, 2,4,6-trimethylphenyl isocyanide (4.0 equiv) for complexes **1**–**3** and (6.0 equiv) for complexes **4**–**5** dissolved in 5 mL of hexane, was added dropwise under stirring. Upon addition, the brown-orange solutions gradually turned light yellow. The reaction mixtures were stirred at room temperature for 1 h. The resulting solids were collected by filtration, washed with hexane (3 × 5 mL).

#### Pathway B

In a glovebox, CuO^*t*^Bu (2.0 equiv) was dissolved in 5 mL of hexane. A solution of ligand [H_2_^2^ **L**], [H_2_^3^ **L**], [H_2_^4^ **L**], [H_2_^6^ **L**], or [H_2_^8^ **L**] (1.00 g, 1.0 equiv) in 5 mL of THF was added dropwise, resulting in a brown-orange solution. After stirring for 15 min at room temperature, 2,4,6-trimethylphenyl isocyanide (2.0 equiv) in 5 mL of hexane was added dropwise. A light-yellow color developed, and the reaction mixture was stirred for 1 h. The product was isolated by filtration and washed with hexane (3 × 5 mL).

[^2^ **L**(Cu-CNR)_2_]: the light-yellow complex [^2^ **L**(Cu-CNR)_2_] was prepared through both synthetic routes (Pathway A and B) and isolated in 83–87% yield. ^1^H NMR (CDCl_3_, 400 MHz, 298 K, δ): 6.88 (s, 4H, Ar-**CH**), 6.10 (s, 2H, β-**H**), 4.22 (s, 4H, N–**CH_2_**), 2.35 (s, 6H, NC**CH_3_**), 3.32 (s, 6H, *ortho*, Ar**CH_3_**), 2.28 (s, 6H, SC**CH_3_**). 2.12 (s, 3H, *para*, Ar**CH_3_**). ^13^C NMR (CDCl_3_, 100 MHz, 298 K, δ): 168.90 (**C**–S), 167.22 (**C**≡N), 139.68 (C≡N–**C**), 134.97 (*ortho*, **C**), 128.51 (*meta*, **C**) 123.20 (*para*, **C),** 119.72 (**C**=N), 58.17 (β-**C**), 34.76. (N–C**C**H_3_), 31.34­(S–C**C**H_3_), 23.64 (N–**C**H_2_), 21.13 (*ortho*, C**C**H_3_),18.61 (*para*, C**C**H_3_).

[^3^ **L**(Cu-CNR)_2_]: the light-yellow complex [^3^ **L**(Cu-CNR)_2_] was prepared through both synthetic routes (Pathway A and B) and isolated in 83–87% yield. ^1^H NMR (CDCl_3_, 400 MHz, 298 K, δ): 6.87 (s, 4H, Ar-**CH**), 6.08 (s, 2H, β-**H**), 3.92–3.88, (t, 4H, *J* = 6 Hz, N–**CH_2_**), 2.35, (s, 6H, NC**CH_3_**), 2.34–2.32 (m, 2H, *J* = 4 Hz, NHCH_2_ **CH_2_**), 3.30 (s, 6H, *ortho*, Ar**CH**_**3**_) 2.28 (s, 6H, SC**CH**_**3**_), 2.05 (s, 3H, *para*, Ar**CH**_**3**_). ^13^C NMR (CDCl_3_, 100 MHz, 298 K, δ): 168.54 (**C**–S), 166.40 (**C**≡N), 139.72 (C≡N–**C**), 134.83 (*ortho*, **C**), 128.83 (*meta*, **C**) 123.96 (*para*, **C),** 120.12 (**C=**N), 54.54 (β-**C**), 35.03 (N–C**C**H_3_), 34.83 (S–C**C**H_3_), 23.54 (N–**C**H_2_), 21.80.81 (N–CH_2_ **C**H_2_), 18.88 (*ortho*, *para* C**C**H_3_).

[^4^ **L**(Cu-CNR)_2_]: the light-yellow complex [^4^ **L**(Cu-CNR)_2_] was prepared through both synthetic routes (Pathway A and B) and isolated in 83–87% yield. ^1^H NMR (CDCl_3_, 400 MHz, 298 K, δ): 6.87 (s, 4H, Ar-**CH**), 6.09 (s, 2H, β-**H**), 3.82–3.79 (t, 4H, *J* = 6 Hz, N–**CH_2_**), 2.37 (s, 6H, NC**CH_3_**), 3.32 (s, 6H, *ortho* Ar**CH_3_**), 2.28, (s, 6H, SC**CH_3_**), 2.04 (s, 3H, *para* Ar**CH_3_**), 1.92–1.88 (qui, 4H, *J* = 4 Hz, NCH_2_ **CH_2_**). ^13^C NMR (CDCl_3_, 100 MHz, 298 K, δ): 168.14 (**C**–S), 165.97 (**C**≡N), 139.84 (C≡N–**C**), 134.89 (*ortho*, **C**), 128.87 (*meta*, **C**) 123.97 (*para*, **C),** 120.17, (**C**=N), 68.11 (β-**C**), 56.77 (N–C**C**H_3_), 35.03 (S–C**C**H_3_), 30.39 (N–**C**H_2_), 23.27 (N–CH_2_ **C**H_2_), 21.41­(*ortho*, C**C**H_3_),18.86 (*para*, C**C**H_3_).

[^6^ **L**(Cu-CNR)_2_]: the light-yellow complex [^6^ **L**(Cu-CNR)_2_] was prepared through both synthetic routes (Pathway A and B), affording light yellow crystals upon recrystallization from toluene at −20 °C in 83–87% yield. ^1^H NMR (CDCl_3_, 400 MHz, 298 K, δ): 6.89 (s, 4H, Ar-**CH**), 6.11 (s, 2H, β-**H**), 3.75–3.72 (t, 4H, *J* = 6.0 Hz, N–**CH_2_**), 2.39 (s, 6H, NC**CH_3_**), 2.33 (s, 6H, *ortho*, Ar**CH_3_**), 2.28 (s, 6H, SC**CH**_**3**_), 2.06 (s, 3H, *para*, Ar**CH**_**3**_), 1.74–1.81 (quentet, 4H, *J* = 5.6 Hz, NCH_2_ **CH_2_**),1.40–1.43 (quented 4H, N­(CH_2_)_2_ **CH_2_CH_2_**). ^13^C NMR (CDCl_3_, 400 MHz, 298 K, δ): 167.94 (**C**–S), 165.71 (**C**≡N), 139.75 (C≡N–**C**), 134.82 (*ortho*, **C**), 128.91 (*meta*, **C**) 124.11 (*para*, **C),** 120.14 (**C**=N), 56.89 (β-**C**), 35.05 (N–C**C**H3), 32.53 (S–C**C**H_3_), 29.85 (N–**C**H_2_), 27.79 (N–CH_2_ **C**H_2_), 23.17 (N-(CH_2_)_2_ **C**H_2_), 21.41 (*ortho*, C**C**H_3_),18.85 (*para*, C**C**H_3_).

[^8^ **L­(**Cu-CNR)_2_]: the light-yellow complex [^8^ **L**(Cu-CNR)_2_] was prepared through both synthetic routes (Pathway A and B), affording light yellow crystals upon recrystallization from toluene at −20 °C in 83–87% yield. ^1^H NMR (CDCl_3_, 400 MHz, 298 K, δ): 6.90 (s, 4H, Ar-**CH**), 6.11 (s, 2H, β-**H**), 3.75–3.71 (t, 4H, *J* = 8.0 Hz, N–**CH_2_**), 2.39 (s, 6H, NC**CH_3_**), 2.33 (s, 6H, *ortho*, Ar**CH_3_**), 2.28 (s, 6H, SC**CH**_**3**_), 2.06 (s, 3H, *para*, Ar**CH**_**3**_), 1.75–73 (m, 4H, *J* = 8.0 Hz, NCH_2_ **CH_2_**),1.35–1.31 (m, 8H, N­(CH_2_)_2_ **CH_2_CH_2_**). ^13^C NMR (CDCl_3_, 100 MHz, 298 K, δ): 167.83 (**C**–S), 165.26 (**C**≡N), 151.86 (C≡N–**C**), 139.63 (*ortho*, **C**), 134.70 (*meta*, **C**) 128.87 (*para*, **C),** 124.18, (**C**=N), 120.20 (β-**C**), 56.93 (N–C**C**H_3_), 35.02 (S–C**C**H_3_), 32.47 (N–**C**H_2_), 29.84 (N–CH_2_ **C**H_2_), 27.63 (N-(CH_2_)_2_ **C**H_2_), 23.20 (N-(CH_2_)_3_ **C**H_2_), 21.40 (*ortho*, C**C**H_3_),18.86 (*para*, C**C**H_3_).

## Supplementary Material



## Notes

Details of structural parameters for complexes **1**, **3–5**, and **9–10**; ^1^H/^13^C NMR, ESI-MS, Raman, FT-IR, UV–vis spectra, and details of ligand synthesis are available in the Supporting Information (PDF)
