# Supplementary material for: Spacer-Length-Dependent Nuclearity and Cuprophilic Modulation in Copper(I) Complexes with Multidentate β‑Thioketiminate Ligands
Source: Inorg Chem. 2026 Jan 9;65(22):12084–93. doi: 10.1021/acs.inorgchem.5c04931 (PMC13250980; doi:10.1021/acs.inorgchem.5c04931)
Supplement: Supplementary file 1 [file ic5c04931_si_001.pdf]

Supporting Information for Spacer-Length-Dependent Nuclearity and Cuprophilic Modulation in Copper(I) Complexes with Multidentate *β*-Thioketiminate Ligands

Najeeb Ullah[a], Venkata Sai Sashankh Penki[a], Yu-Ting Chu[b], Amir Karim[a], Rahime Eshaghi Malekshah[a], Sodio C.N. Hsu[a, c*]

[a] Department of Medicinal and Applied Chemistry, Kaohsiung Medical University, Kaohsiung 80708, Taiwan
[b] International PhD Program for Science, National Sun Yat-Sen University, Kaohsiung 80059, Taiwan
[c] Department of Medical Research, Kaohsiung Medical University Hospital, Kaohsiung 80708, Taiwan
Corresponding author: email: sodiohsu@kmu.edu.tw

Table of contents

Ligand Synthesis……………………………………………………………….……………S4-S5

Figure S1. ^{1}H NMR spectra of $H_2{}^2$**L** in $CDCl_3$ (400 MHz, 298 K). Solvent residual peaks are marked with an asterisk (*)…………………………………...………..……………………S6

Figure S2. ^{1}H NMR spectra of $H_2{}^3$**L** in $CDCl_3$(400 MHz, 298 K). Solvent residual peaks are marked with an asterisk (*)……………………………………………………………………….S6

Figure S3. ^{1}H NMR spectra of $H_2{}^4$**L** in $CDCl_3$ (400 MHz, 298 K). Solvent residual peaks are marked with an asterisk (*)………………………..…………………………………………….…..S7

Figure S4. ^{1}H NMR spectra of $H_2{}^6$**L** in $CDCl_3$ (400 MHz, 298 K). Solvent residual peaks are marked with an asterisk (*)………………………..……………………………………….……..S7

Figure S5. ^{1}H NMR spectra of $H_2{}^8$**L** in $CDCl_3$ (400 MHz, 298 K). Solvent residual peaks are marked with an asterisk (*)………………………..………………………………………….……..S8

Figure S6. ^{13}C NMR spectra of [$H_2{}^2$**L**] in $CDCl_3$(100 MHz, 298 K). Solvent residual peaks are marked with an asterisk (*)………………………………………………………..……...……..S8

Figure S7. ^{13}C NMR spectra of [$H_2{}^3$**L**] in $CDCl_3$(100 MHz, 298 K). Solvent residual peaks are marked with an asterisk (*)………………………………………………………..……...……..S9

Figure S8. ^{13}C NMR spectra of [$H_2{}^4$**L**] in $CDCl_3$(100 MHz, 298 K). Solvent residual peaks are marked with an asterisk (*)………………………………………………………..……...……..S9

Figure S9. ^{13}C NMR spectra of [$H_2{}^6$**L**] in $CDCl_3$(100 MHz, 298 K). Solvent residual peaks are marked with an asterisk (*)……………………………………………………………………S10
Figure S10. ^{13}C NMR spectra of [$H_2{}^8$**L**] in $CDCl_3$(100 MHz, 298 K). Solvent residual peaks are marked with an asterisk (*)……………………………………………………………………S10
Figure S11. ^{1}H NMR spectra of [^{2}**L**Cu_2]$_2$ in $CDCl_3$(400 MHz, 298 K). Solvent residual peaks are marked with an asterisk (*) ……………………………………………………………………S11
Figure S12. ^{1}H NMR spectra of [^{3}**L**Cu_2]$_2$ in $CDCl_3$(400 MHz, 298 K). Solvent residual peaks are marked with an asterisk (*)……………………………………………………………………S11
Figure S13. ^{1}H NMR spectra of [^{4}**L**Cu_2]$_2$ in $CDCl_3$(400 MHz, 298 K). Solvent residual peaks are marked with an asterisk (*)……………………………………………………………………S12
Figure S14. ^{1}H NMR spectra of [^{6}**L**Cu_2]$_3$ in CD_2Cl_2 (400 MHz, 298 K). Solvent residual peaks are marked with an asterisk (*)……………………………………………………………………S12
Figure S15. ^{1}H NMR spectra of [^{8}**L**Cu_2]$_3$ in CD_2Cl_2 (400 MHz, 298 K). Solvent residual peaks are marked with an asterisk (*)……………………………………………………………………S13
Figure S16. ^{13}C NMR spectra of [^{2}**L**Cu_2]$_2$ in $CDCl_3$ (100 MHz, 298 K). Solvent residual peaks are marked with an asterisk (*)……………………………………………………………………S13
Figure S17. ^{13}C NMR spectra of [^{3}**L**Cu_2]$_2$ in $CDCl_3$ (100 MHz, 298 K). Solvent residual peaks are marked with an asterisk (*)……………………………………………………………………S14
Figure S18. ^{13}C NMR spectra of [^{4}**L**Cu_2]$_2$ in $CDCl_3$ (100 MHz, 298 K). Solvent residual peaks are marked with an asterisk (*)……………………………………………………………………S14
Figure S19. ^{13}C NMR spectra of [^{6}**L**Cu_2]$_3$ in CD_2Cl_2 (100 MHz, 298 K). Solvent residual peaks are marked with an asterisk (*)……………………………………………………………………S15
Figure S20. ^{13}C NMR spectra of [^{8}**L**Cu_2]$_3$ in CD_2Cl_2 (100 MHz, 298 K). Solvent residual peaks are marked with an asterisk (*)……………………………………………………………………S15
Figure S21. ^{1}H NMR spectra of [^{2}**L**(Cu-CNR)$_2$] in $CDCl_3$ (400 MHz, 298 K). Solvent residual peaks are marked with an asterisk (*) ……………………………………………………………………S16
Figure S22. ^{1}H NMR spectra of [^{3}**L**(Cu-CNR)$_2$] in $CDCl_3$(400 MHz, 298 K). Solvent residual peaks are marked with an asterisk (*)……………………………………………………………………S16
Figure S23. ^{1}H NMR spectra of [^{4}**L**(Cu-CNR)$_2$] in $CDCl_3$(400 MHz, 298 K). Solvent residual peaks are marked with an asterisk (*)……………………………………………………………………S17

Figure S24. ^{1}H NMR spectra of [^{6}**L**(Cu-CNR)$_2$] in $CDCl_3$(400 MHz, 298 K). Solvent residual peaks are marked with an asterisk (*)……………………………………..……..……..………S17
Figure S25. ^{1}H NMR spectra of [^{8}**L**(Cu-CNR)$_2$] in $CDCl_3$(400 MHz, 298 K). Solvent residual peaks are marked with an asterisk (*)……………………………………..……..……..………S18
Figure S26. ^{13}C NMR spectra of [^{2}**L**(Cu-CNR)$_2$] in $CDCl_3$ (100 MHz, 298 K). Solvent residual peaks are marked with an asterisk (*)………………………………..…………..…………S18
Figure S27. ^{13}C NMR spectra of [^{3}**L**(Cu-CNR)$_2$] in $CDCl_3$ (100 MHz, 298 K). Solvent residual peaks are marked with an asterisk (*)………………………………..…………..…………S19
Figure S28. ^{13}C NMR spectra of [^{4}**L**(Cu-CNR)$_2$] in $CDCl_3$ (100 MHz, 298 K). Solvent residual peaks are marked with an asterisk (*)………………………………..…………..…………S19
Figure S29. ^{13}C NMR spectra of [^{6}**L**(Cu-CNR)$_2$] in $CDCl_3$ (100 MHz, 298 K). Solvent residual peaks are marked with an asterisk (*)……………………………..………..…..…………S20
Figure S30. ^{13}C NMR spectra of [^{8}**L**(Cu-CNR)$_2$] in $CDCl_3$ (100 MHz, 298 K). Solvent residual peaks are marked with an asterisk (*)……………………………..……..……..…………S20
Figure S31. UV-Vis spectra of the thiolate ligands (H^{1}**L**-H^{4}**L**) with corresponding copper(I) thiolate complexes [^{n}**L**Cu$_2$]$_2$, and [^{n}**L**Cu$_2$]$_3$………………………..……..…………………..S21
Figure S32. Raman spectra of [**L**Cu$_2$]$_2$, complexes (red) and their corresponding isocyanide adducts [**L**(Cu-CNR)$_2$] (blue)………..……………………………………………..……..……. S21
Figure S33. Raman spectra of [**L**Cu$_2$]$_3$, complexes (red) and their corresponding isocyanide adducts [**L**(Cu-CNR)$_2$] (blue)…………………………………………………………………..S22
Figure S34. ESI-MS spectra of the thiolate ligand H$_2$^{2}**L**………………….........……………..S22
Figure S35. ESI-MS spectra of the thiolate ligand H$_2$^{3}**L**. . ……………………….……………S23
Figure S36. ESI-MS spectra of the thiolate ligand H$_2$^{4}**L** ……………..……..…..……………S23
Figure S37. ESI-MS spectra of the thiolate ligand H$_2$^{6}**L** ……………..…..……...……………S24
Figure S38. ESI-MS spectra of the thiolate ligand H$_2$^{8}**L**. …….………….……………...…….S24
Figure S39. FT-IR spectra of (Mesityl-NC) and its Copper(I) complexes. ….….……………….S25
Figure S40. FT-IR spectra of ligand H$_2$^{2}**L** and its corresponding copper(I) complex [^{2}**L**Cu$_2$]$_2$. ………S25
Table S1. Crystal data and structure refinement parameters for the Cu(I) β-thioketiminate complexes…………………………………………………………………………………….……….26
Table S2. Selected bond distances (Å) and bond angles (°) for copper(I) complexes [^{2}**L**Cu$_2$]$_2$, [^{4}**L**Cu$_2$]$_2$, [^{6}**L**Cu$_2$]$_3$ and [^{8}**L**Cu$_2$]$_3$…………………………………………………………27

Table S3. Selected bond distances (Å) and bond angles (°) for copper(I) complexes [^{6}**L**(Cu-CNR)$_2$], and [^{8}**L**(Cu-CNR)$_2$]. . ……………………..………..…….……...……………………..……………………28

Ligand Synthesis:

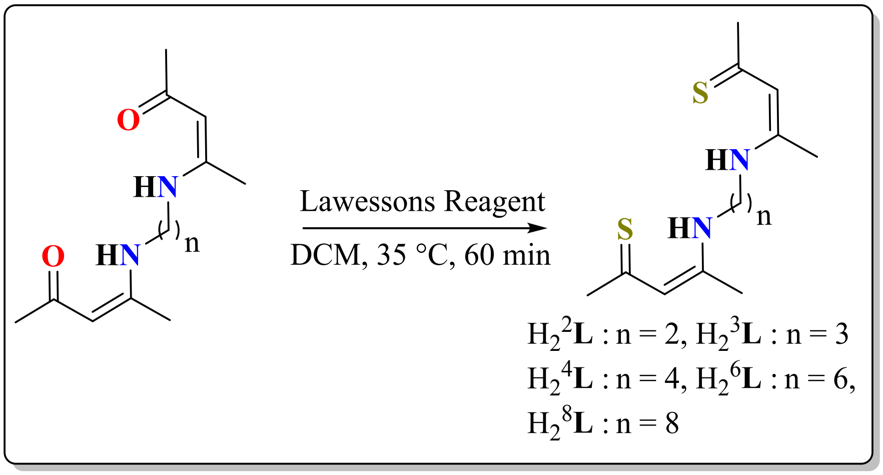

[H_2^{2}**L**]. (3Z,3'Z)-4,4'-(ethane-1,2-diylbis(azanediyl))bis(pent-3-en-2-one) (5.00 g, 0.022 mol) and an equimolar amount of Lawesson's reagent (9.00 g, 0.022 mol) were dissolved in 0.05 L of dichloromethane. The reaction mixture was stirred and heated at 35 °C for 1 hour. After completion, the solvent was removed under reduced pressure to afford a yellow-radish powder. The crude product was purified by column chromatography using a DCM/hexane (3:1) eluent system. The desired compound was obtained as a pure yellow powder (4.83 g, 86% yield). ^{1}H NMR ($CDCl_3$, 400 MHz, 298 K, δ): 14.24 (s, 2H, **N*H***), 6.12 (s, 2H, ***β-H***), 3.73-3.72 (t, 4H, J = 4.0 Hz, N-**C*H*$_2$**), 2.53 (s, 6H, NC**C*H*$_3$**), 2.10 (s, 6H, SC**C*H*$_3$**). ^{13}C ($CDCl_3$, 400 MHz, 298 K, δ): 195.67 (***C***=S), 163.04 (C=***C***-N), 96.27 (***β-C***), 43.60 (N-**C**H$_2$), 28.94 (NC***C***H$_3$), 18.79 (SC***C***H$_3$). Uv-vis 390 nm (ε= 30,800 $M^{-1}\cdot cm^{-1}$). **ESI-MS** (MeOH), negative mode: calcd for [H_2^{8}**L**-H^+]$^-$ 256.11; found =255.19 (Figure S34).

[H_2^{3}**L**]. (3Z,3'Z)-4,4'-(propane-1,3-diylbis(azanediyl))bis(pent-3-en-2-one) (5.00 g, 0.020 mol) and an equimolar amount of Lawesson's reagent (8.48 g, 0.020 mol) were dissolved in 0.05 L of dichloromethane. The reaction mixture was stirred and heated at 35 °C for 1 hour. After completion, the solvent was removed under reduced pressure to afford a yellow-radish powder. The crude product was purified by column chromatography using a DCM/hexane (3:1) eluent system. The desired compound was obtained as a pure yellow powder (4.77 g, 88% yield). 1H NMR ($CDCl_3$, 400 MHz, 298 K, δ): 14.15 (s, 2H, **N*H***), 6.13 (s, 2H, ***β-H***), 3.68-3.63 (m, 4H, J = 6.66 Hz, N- **C*H*$_2$**), 2.51 (s, 6H, NC**C*H*$_3$**), 3.14-2.07 (q, 4H, J = 7.0 Hz, NHCH$_2$**C*H*$_2$**), 2.10 (s, 6H, SC**C*H*$_3$**).^{13}C NMR ($CDCl_3$, 400 MHz, 298 K, δ): *203*.72 (***C***=S), 166.30 (C=***C***-

N), 113.49 (*β-**C***), 43.45 (N-**C**H_2), 38.62 (NC***C***H_3) 26.72 (SC**C**H_3), 20.62 ($NHCH_2$***C***H_2). 384 nm **(**ε= 25,120 $M^{-1}\cdot cm^{-1}$). **ESI-MS** (MeOH), negative mode: calcd for $[H_2{}^8$**L**$\text{-}H^+]^-$ 270.12; found =269.20 (Figure S35).

$[H_2{}^4$**L**]. (3Z,3'Z)-4,4'-(butane-1,4-diylbis(azanediyl))bis(pent-3-en-2-one) (5.00 g, 0.019 mol) and an equimolar amount of Lawesson's reagent (8.00 g, 0.019 mol) were dissolved in 0.05 L of dichloromethane. The reaction mixture was stirred and heated at 35 °C for 1 hour. After completion, the solvent was removed under reduced pressure to afford a yellow-radish powder. The crude product was purified by column chromatography using a DCM/hexane (3:1) eluent system. The desired compound was obtained as a pure yellow powder (4.62 g, 85% yield). ^{1}H NMR ($CDCl_3$, 400 MHz, 298 K, δ): 14.13 (s, 2H, **N*H***), 6.12 (s, 2H, ***β-H***), 3.49-3.45 (m, 4H, J = 5.33 Hz, N-**C*H*$_2$**), 2.51 (s, 6H, NC**C*H*$_3$**), 2.09 (s, 6H, SC**C*H*$_3$**), 1.91-1.88 (q, 4H, J = 5.33 Hz, $NHCH_2$**C*H*$_2$**). ^{13}C NMR ($CDCl_3$, 400 MHz, 298 K, δ): *203*.74 (***C***=S), 166.30 (C=***C***-N), 113.49 (*β-**C***), 43.50 (N-***C***H_2), 38.63 (NC***C***H_3) 26.72 (SC***C***H_3), 20.62 (N-CH_2***C***H_2). 383 nm **(**ε= 25,640 $M^{-1}\cdot cm^{-1}$**)**. **ESI-MS** (MeOH), negative mode: calcd for $[H_2{}^4$**L**$\text{-}H^+]^-$ 284.14; found =283.21 (Figure S36).

$[H_2{}^6$**L**]. (3Z,3'Z)-4,4'-(hexane-1,6-diylbis(azanediyl))bis(pent-3-en-2-one) (5.00 g, 0.017 mol) and an equimolar amount of Lawesson's reagent (7.20 g, 0.017 mol) were dissolved in 0.05 L of dichloromethane. The reaction mixture was stirred and heated at 35 °C for 1 hour. After completion, the solvent was removed under reduced pressure to afford a yellow-radish powder. The crude product was purified by column chromatography using a DCM/hexane (3:1) eluent system. The desired compound was obtained as a pure yellow powder (4.60 g, 86% yield). ^{1}H NMR ($CDCl_3$, 400 MHz, 298 K, δ): 14.03 (s, 2H, **N*H***), 6.09 (s, 2H, ***β-H***), 3.42-3.37 (m, 4H, J = 6.68 Hz, N-**C*H*$_2$**), 2.49 (s, 6H, NC**C*H*$_3$**), 2.06 (s, 6H, SC**C*H*$_3$**), 1.74-1.68 (q, 4H, J = 6.00 Hz, $NHCH_2$**C*H*$_2$**), 1.53-1.50 (q, 4H, J = 3.00 Hz, $NH(CH_2)_2$**C*H*$_2$**),. ^{13}C NMR ($CDCl_3$, 400 MHz, 298 K, δ): *202*.61 (***C***=S), 166.36 (C=***C***-N), 113.44 (*β-**C***), 43.76 (N-***C***H_2), 38.52 (NC***C***H_3), 28.77 ($NHCH_2$***C***H_2), 26.37 (SC***C***H_3), 20.61 ($NHC(CH_2)_2$***C***H_2). 382 nm **(**ε = 32,760 $M^{-1}\cdot cm^{-1}$**)**. **ESI-MS** (MeOH), negative mode: calcd for $[H_2{}^6$**L**$\text{-}H^+]^-$ 312.17; found =311.30 (Figure S37).

$[H_2{}^8$**L**]. (3Z,3'Z)-4,4'-(octane-1,8-diylbis(azanediyl))bis(pent-3-en-2-one) (5.00 g, 0.016 mmol) and an equimolar amount of Lawesson's reagent (6.46 g, 0.016 mmol) were dissolved in 50 mL of dichloromethane. The reaction mixture was stirred and heated at 35 °C for 1 hour. After completion, the solvent was removed under reduced pressure to afford a yellow-radish powder. The crude product was purified by column chromatography using a DCM/hexane (3:1) eluent system. The desired compound was obtained as a pure yellow powder (4.72 g, 87% yield). ^{1}H NMR ($CDCl_3$, 400 MHz, 298 K, δ): 14.05 (s, 2H, **N*H***), 6.10 (s, 2H, ***β-H***), 3.41-3.36 (m, 4H, J = 6.66 Hz, N-**C*H*$_2$**), 2.51 (s, 6H, NC**C*H*$_3$**), 2.07 (s, 6H, SC**C*H*$_3$**), 1.73-1.66 (q, 4H, J = 7 Hz, $NHCH_2$**CH_2**), 1.50-1.43 (q, 4H, J = 7 Hz, $NHCH_2CH_2$**C*H*$_2$**C*H*$_2$), 1.38-1.34 (q,

4H, J = 4.0 Hz, $NHCH_2CH_2CH_2\boldsymbol{CH_2}$). ^{13}C NMR ($CDCl_3$, 400 MHz, 298 K, δ): *203*.04 (***C***=S), 166.12 (C=***C***-N), 113.38 (*β*-***C***), 44.03 (N-***C***H_2), 38.59 (NC***C***H_3) 29.15 (SC***C***H3), 28.90 ($NHCH_2$***C***H_2), 26.83 ($NH(CH_2)_2$***C***H_2), 20.59 ($NH(CH_2)_3$***C***H_2). 382 nm **(**ε = 31,640 $M^{-1}\cdot cm^{-1}$). **ESI-MS** (MeOH), negative mode: calcd for $[H_2{}^8\mathbf{L}\text{-}H^+]^-$ 340.20; found =339.32 (Figure S38).

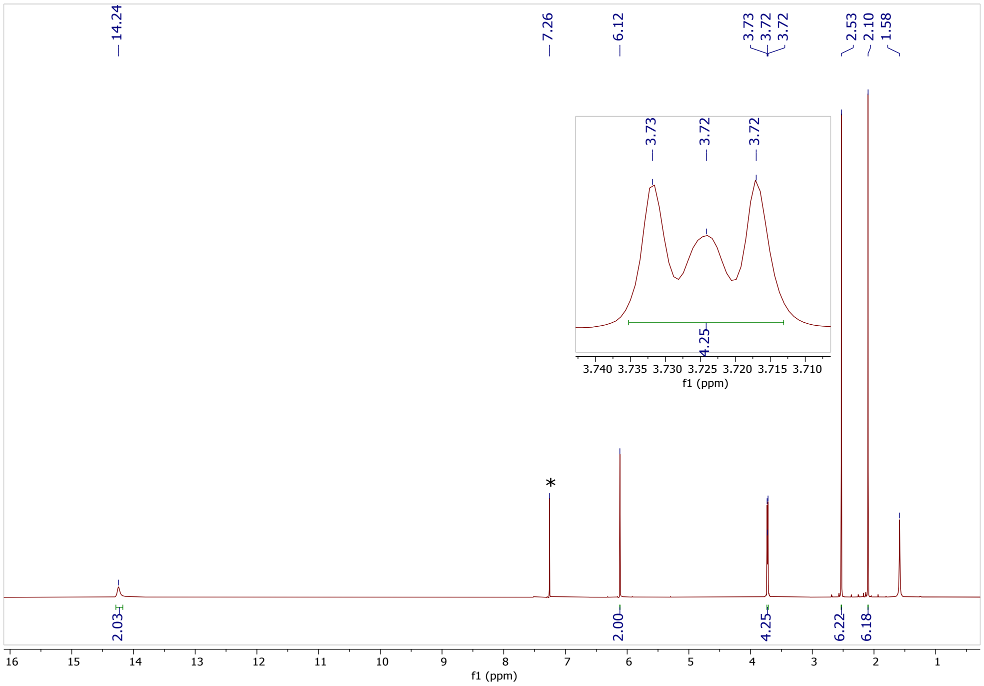

Figure S1. ^{1}H NMR spectra of $H_2{}^2\mathbf{L}$ in $CDCl_3$ (400 MHz, 298 K). Solvent residual peaks are marked with an asterisk (*).

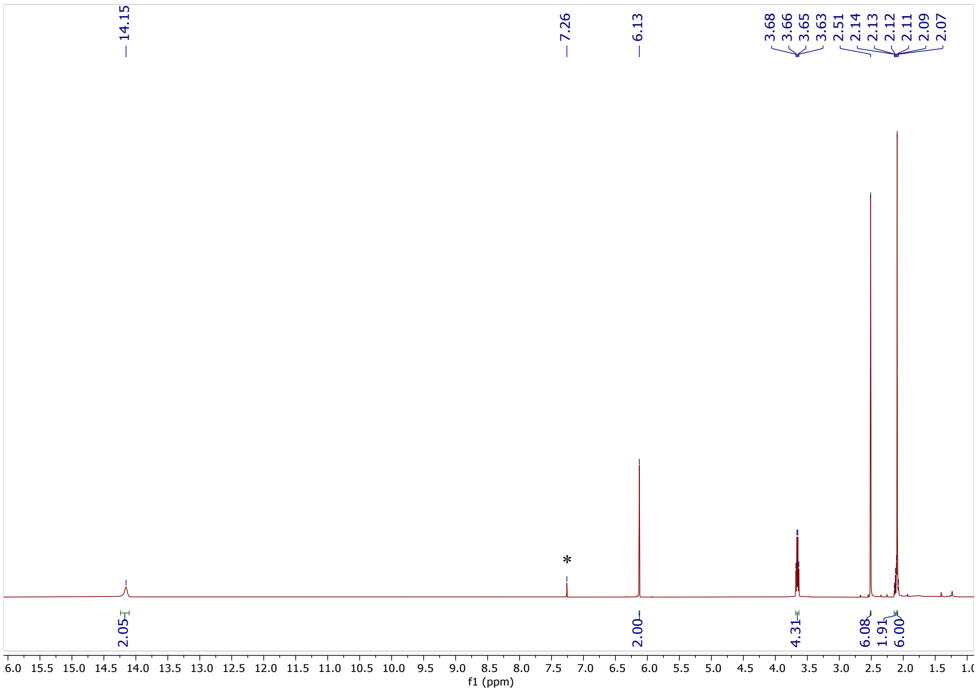

Figure S2. ^{1}H NMR spectra of $H_2{}^{3}\mathbf{L}$ in $CDCl_3$ (400 MHz, 298 K). Solvent residual peaks are marked with an asterisk (*).

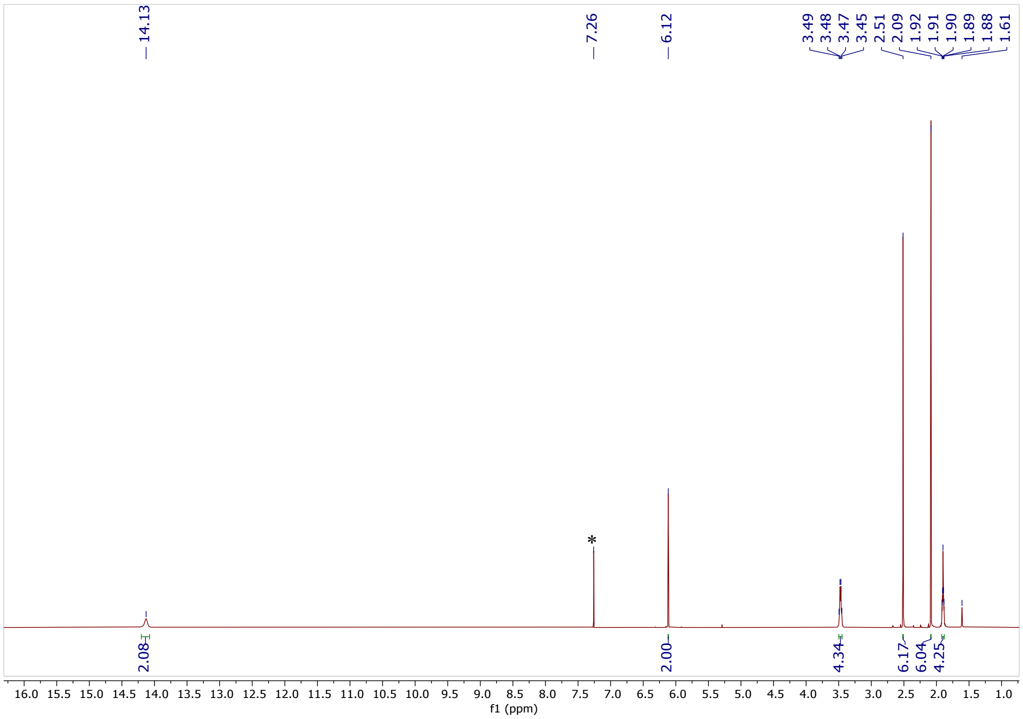

Figure S3. ^{1}H NMR spectra of $H_2{}^{4}\mathbf{L}$ in $CDCl_3$ (400 MHz, 298 K). Solvent residual peaks are marked with an asterisk (*).

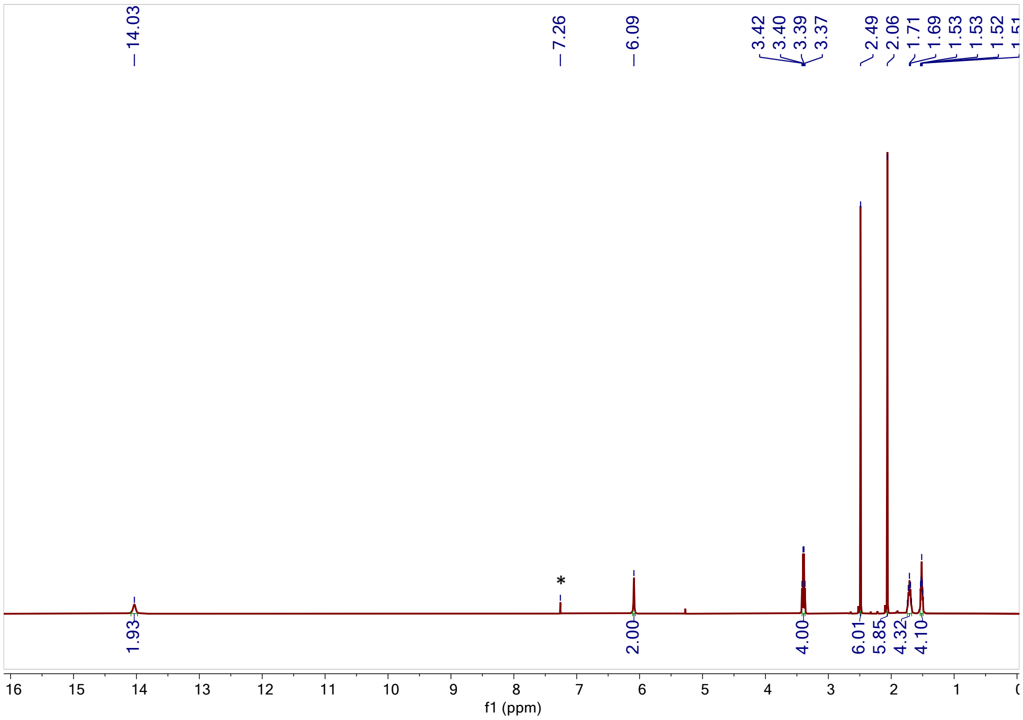

Figure S4. ^{1}H NMR spectra of $H_2{}^6$**L** in $CDCl_3$ (400 MHz, 298 K). Solvent residual peaks are marked with an asterisk (*).

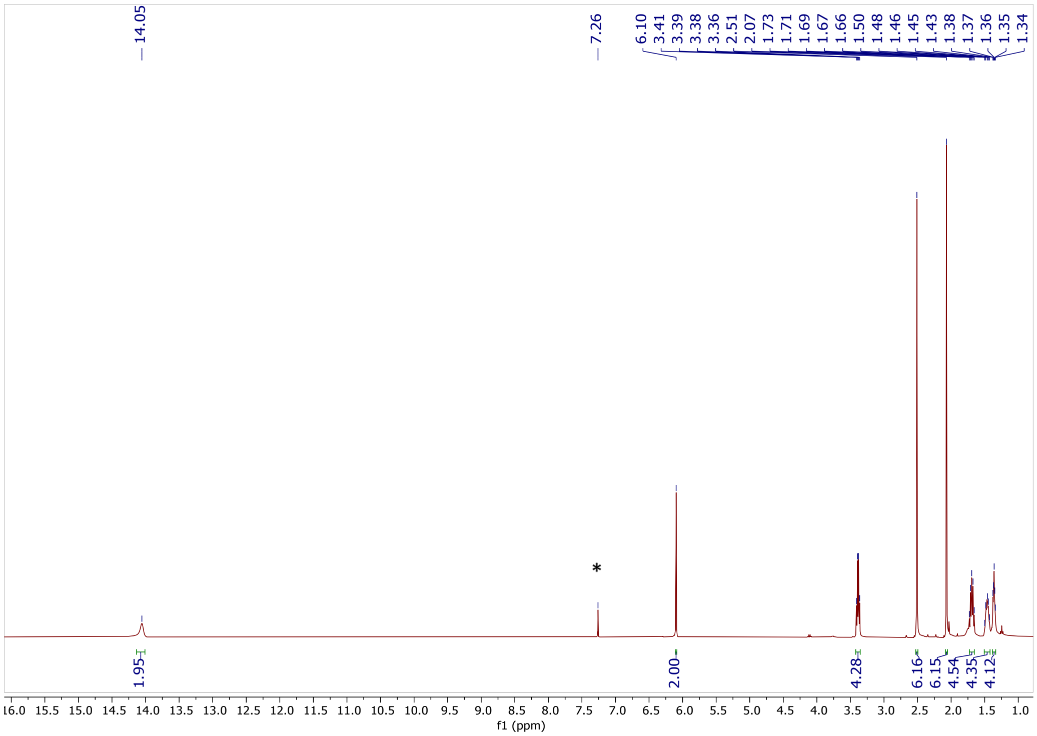

Figure S5. ^{1}H NMR spectra of $H_2{}^8$**L** in $CDCl_3$ (400 MHz, 298 K). Solvent residual peaks are marked with an asterisk (*).

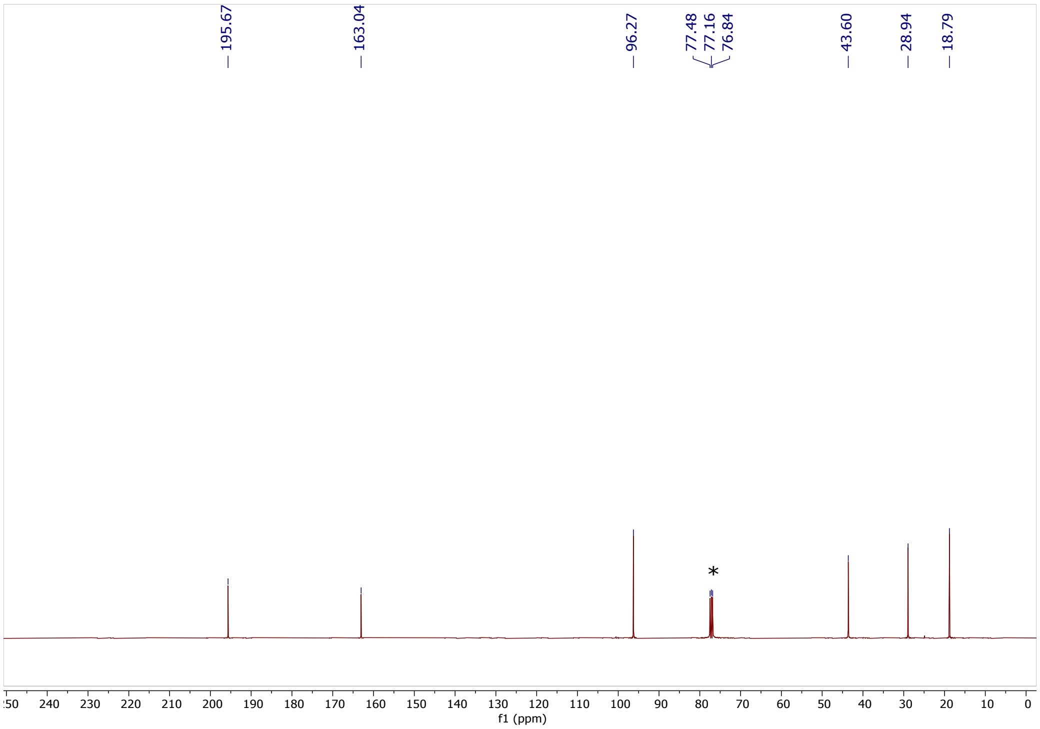

Figure S6. ^{13}C NMR spectra of [H_2^{2}**L**] in $CDCl_3$ (400 MHz, 298 K). Solvent residual peaks are marked with an asterisk (*).

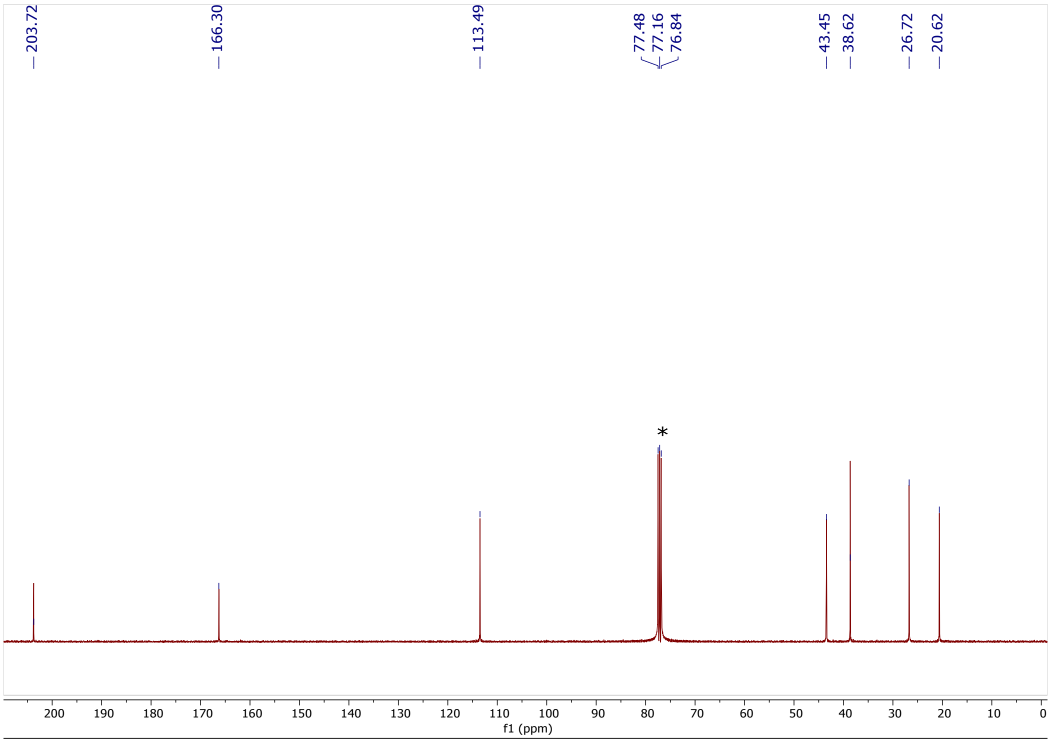

Figure S7. ^{13}C NMR spectra of H_2^{3}**L** in $CDCl_3$ (100 MHz, 298 K). Solvent residual peaks are marked with an asterisk (*).

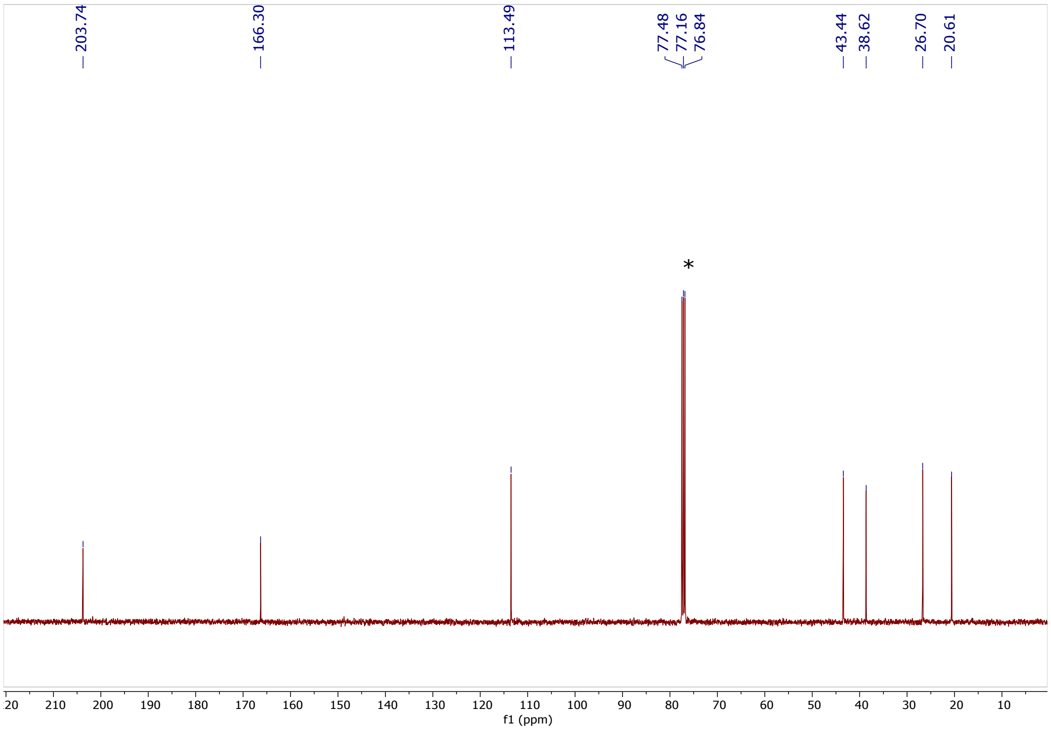

Figure S8. ^{13}C NMR spectra of $H_2{}^4\mathbf{L}$ in $CDCl_3$ (100 MHz, 298 K). Solvent residual peaks are marked with an asterisk (*).

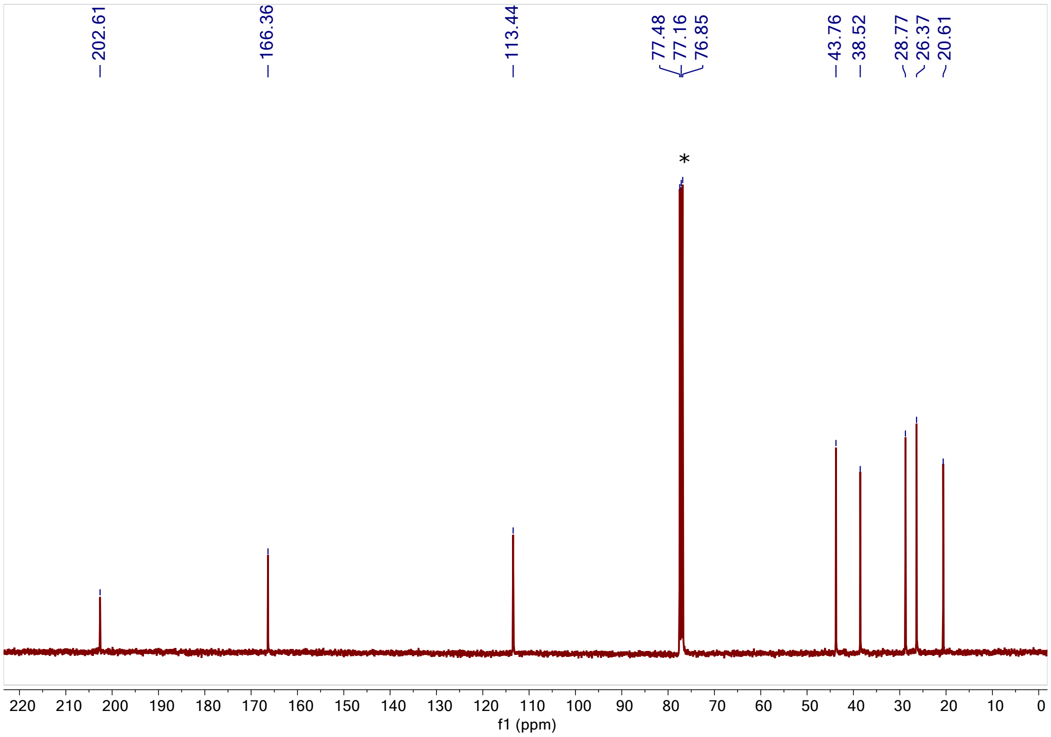

Figure S9. ^{13}C NMR spectra of $H_2{}^6\mathbf{L}$ in $CDCl_3$ (100 MHz, 298 K). Solvent residual peaks are marked with an asterisk (*).

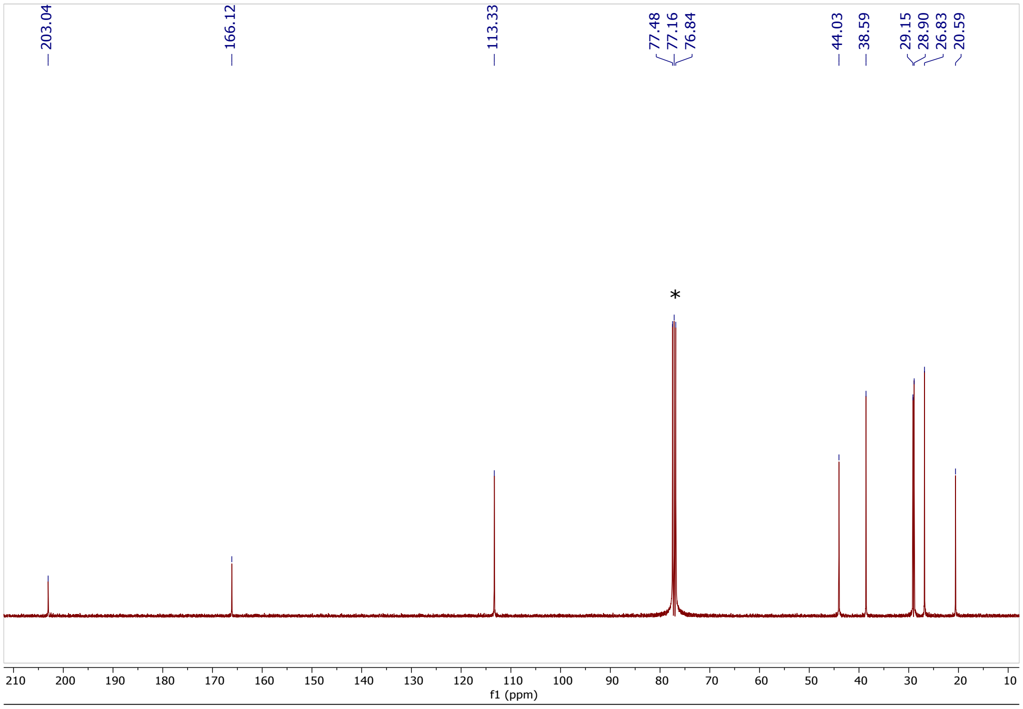

Figure S10. ^{13}C NMR spectra of $H_2{}^8$**L** in $CDCl_3$ (100 MHz, 298 K). Solvent residual peaks are marked with an asterisk (*).

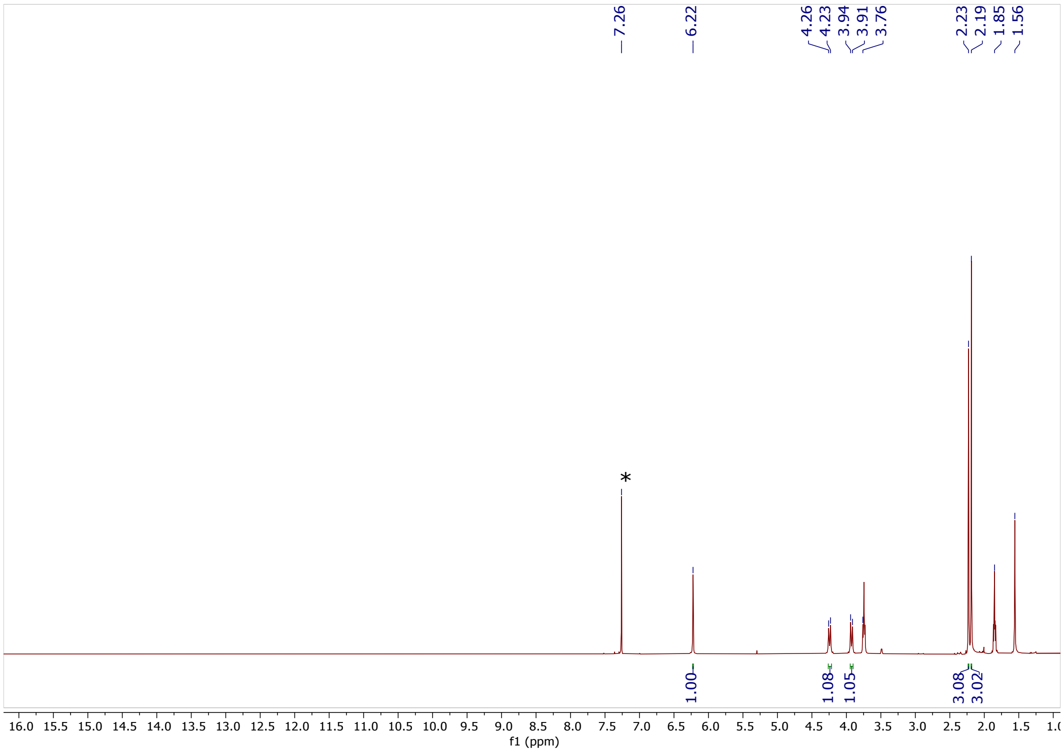

Figure S11. ^1H NMR spectra of $[^2\mathbf{L}Cu_2]_2$ in $CDCl_3$ (100 MHz, 298 K). Solvent residual peaks are marked with an asterisk (*).

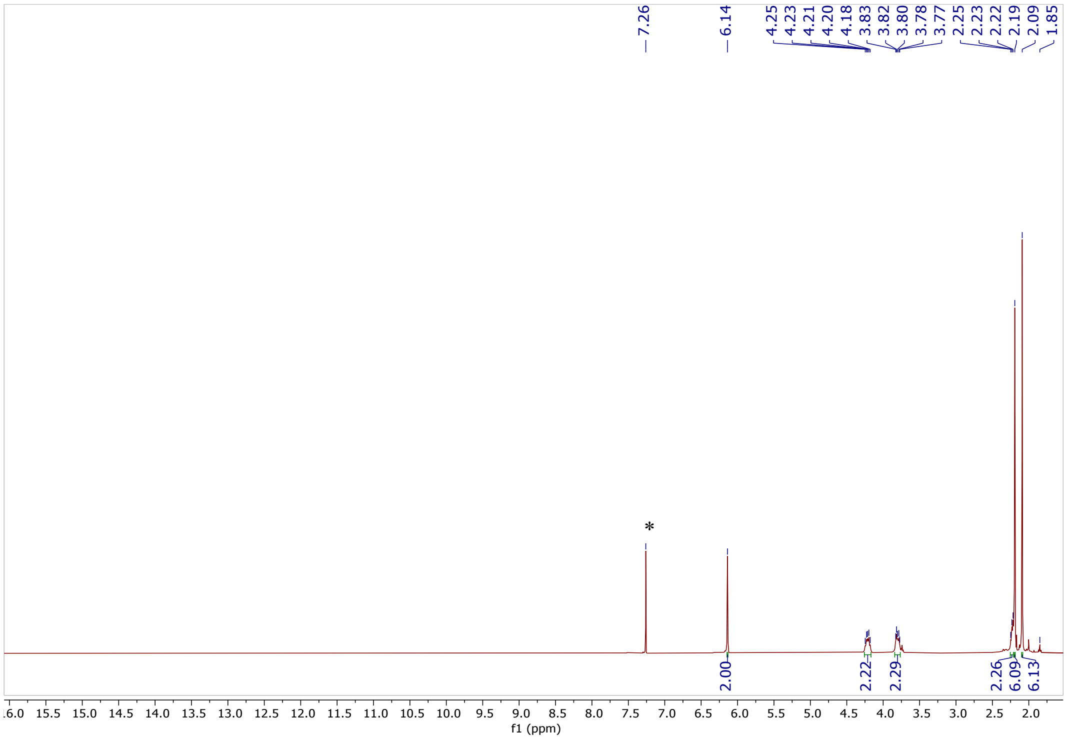

Figure S12. ^{1}H NMR spectra of $[^3\mathbf{L}Cu_2]_2$ in $CDCl_3$ (100 MHz, 298 K). Solvent residual peaks are marked with an asterisk (*).

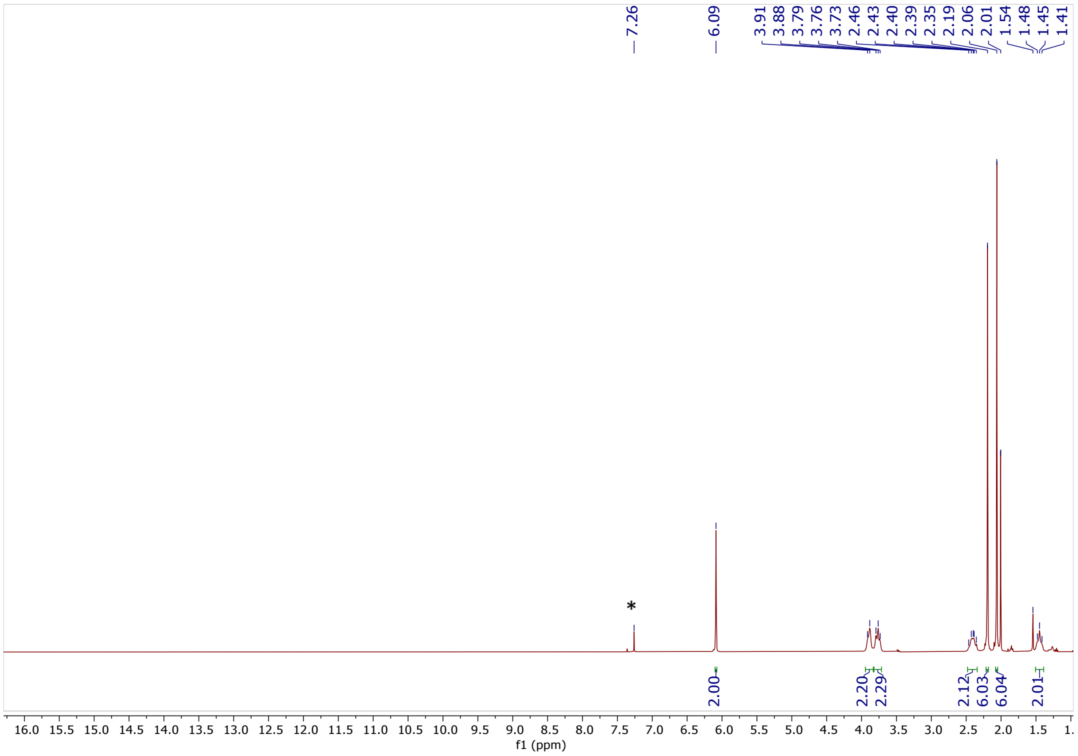

Figure S13. ^{1}H NMR spectra of $[^4\mathbf{L}Cu_2]_2$ in $CDCl_3$ (400 MHz, 298 K). Solvent residual peaks are marked with an asterisk (*).

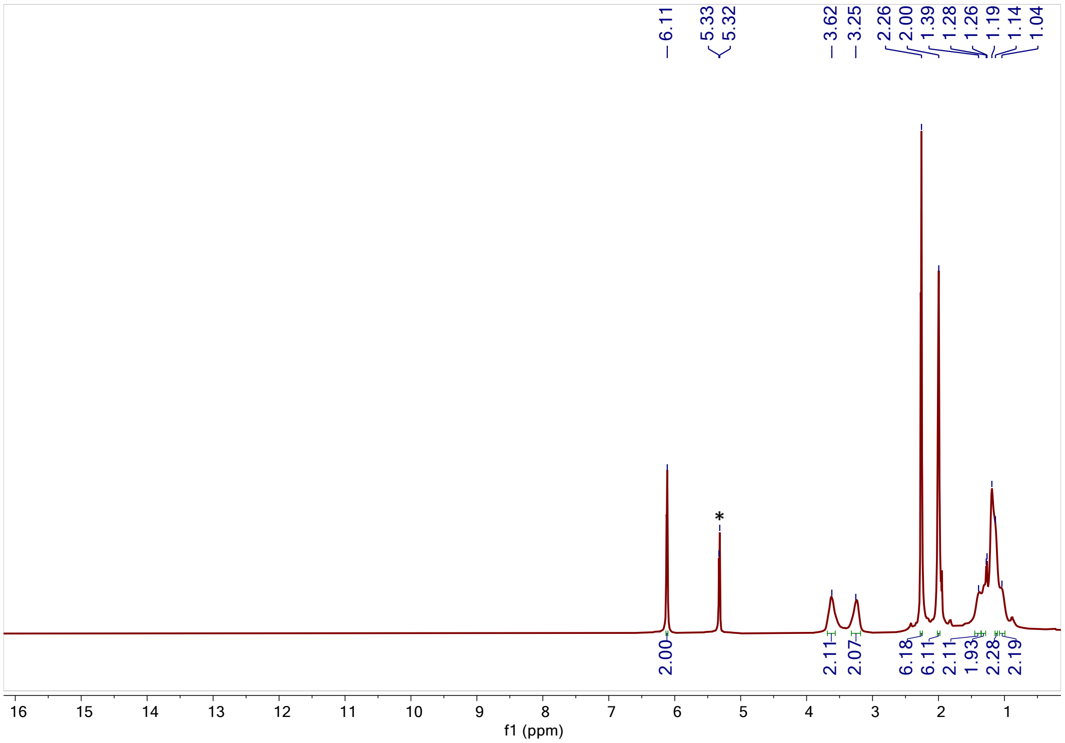

Figure S14. ^{1}H NMR spectra of $[^{6}\mathbf{L}Cu_2]_3$ in CD_2Cl_2 (400 MHz, 298 K). Solvent residual peaks are marked with an asterisk (*).

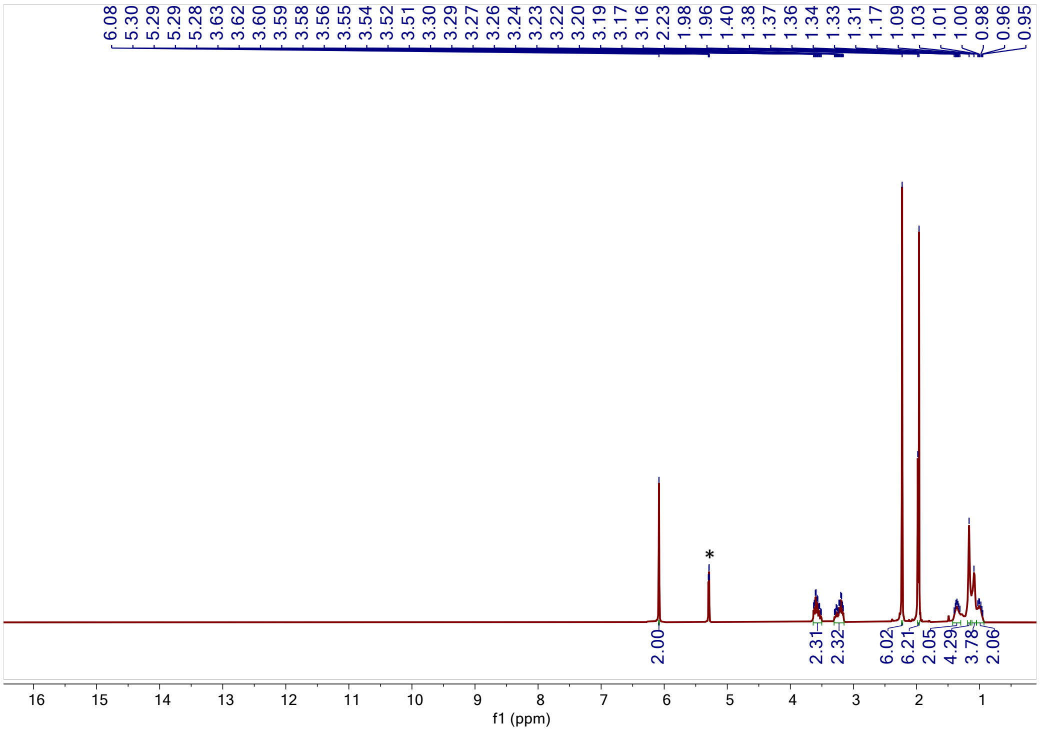

Figure S15. ^{1}H NMR spectra of $[^{8}\mathbf{L}Cu_2]_3$ in CD_2Cl_2 (400 MHz, 298 K). Solvent residual peaks are marked with an asterisk (*).

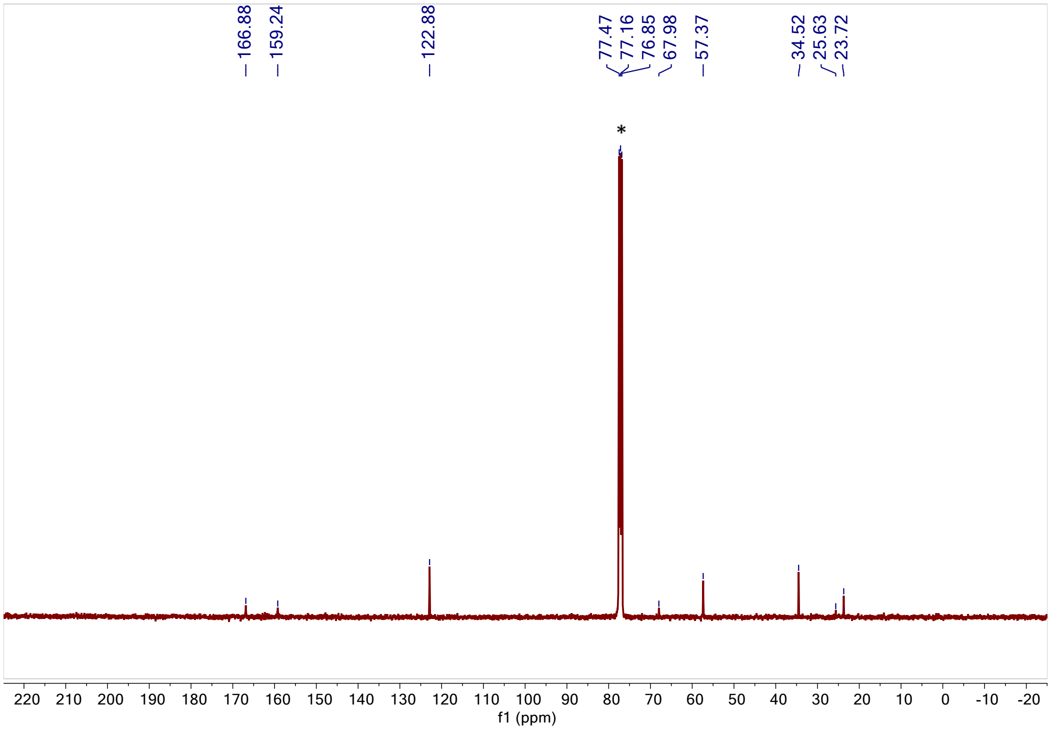

Figure S16. ^{13}C NMR spectra of $[^{2}\mathbf{L}Cu_2]_2$ in $CDCl_3$ (100 MHz, 298 K). Solvent residual peaks are marked with an asterisk (*).

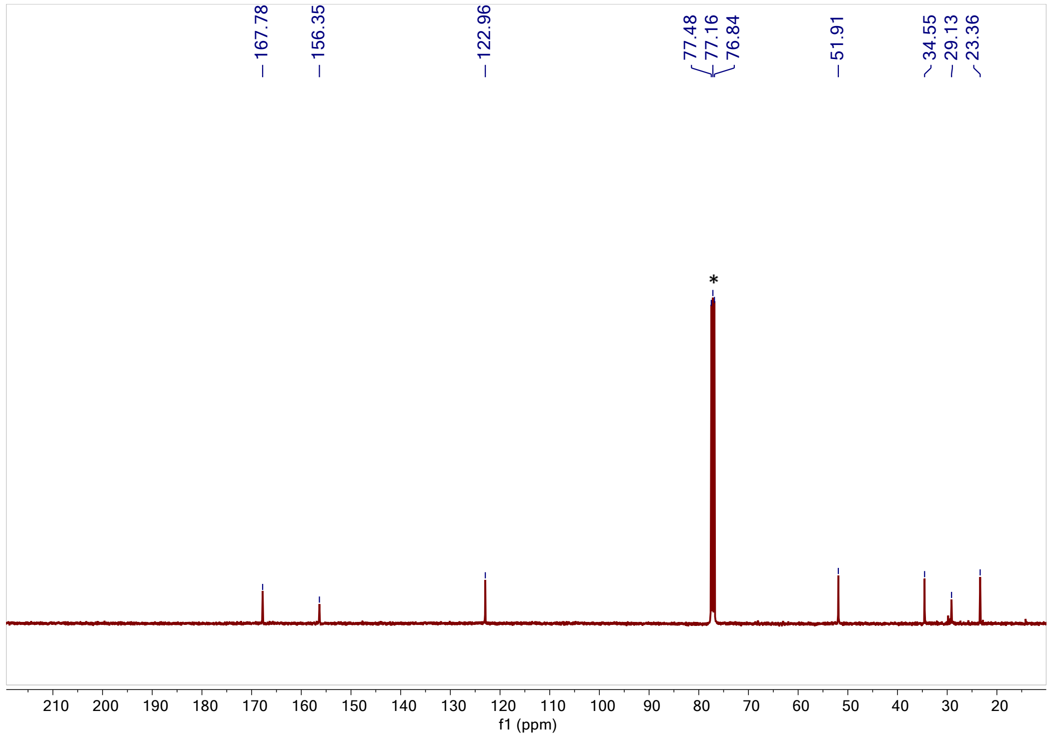

Figure S17. ^{13}C NMR spectra of $[^{3}\mathbf{L}Cu_2]_2$ in $CDCl_3$ (100 MHz, 298 K). Solvent residual peaks are marked with an asterisk (*).

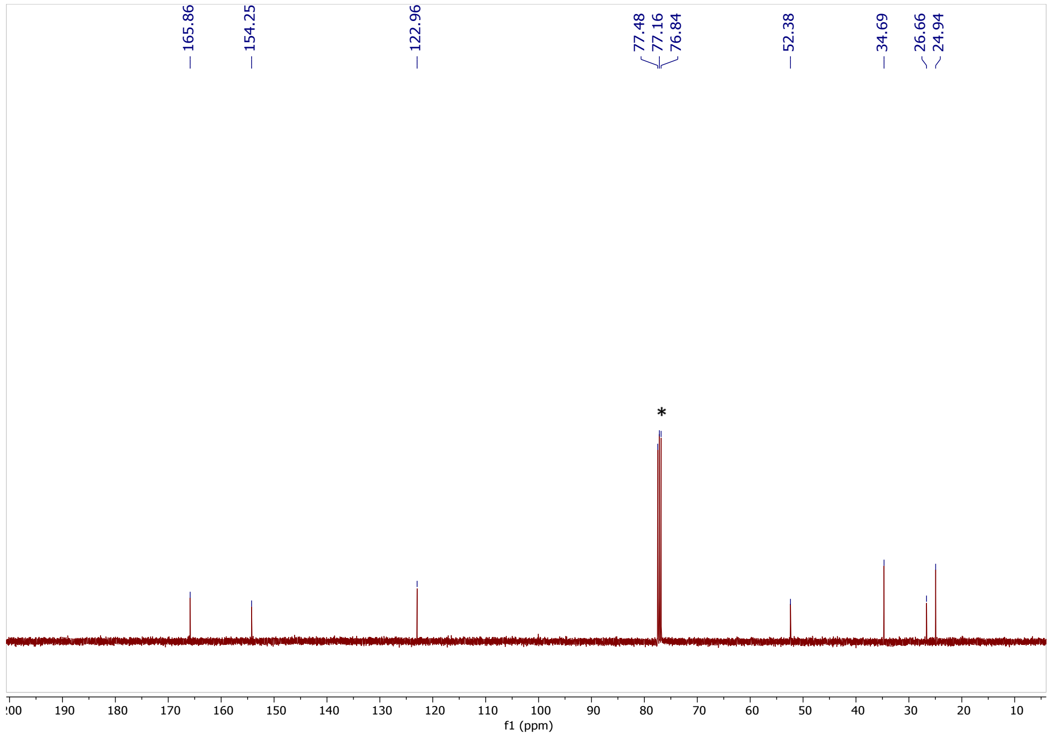

Figure S18. ^{13}C NMR spectra of $[^{4}\mathbf{L}Cu_2]_2$ in $CDCl_3$ (100 MHz, 298 K). Solvent residual peaks are marked with an asterisk (*).

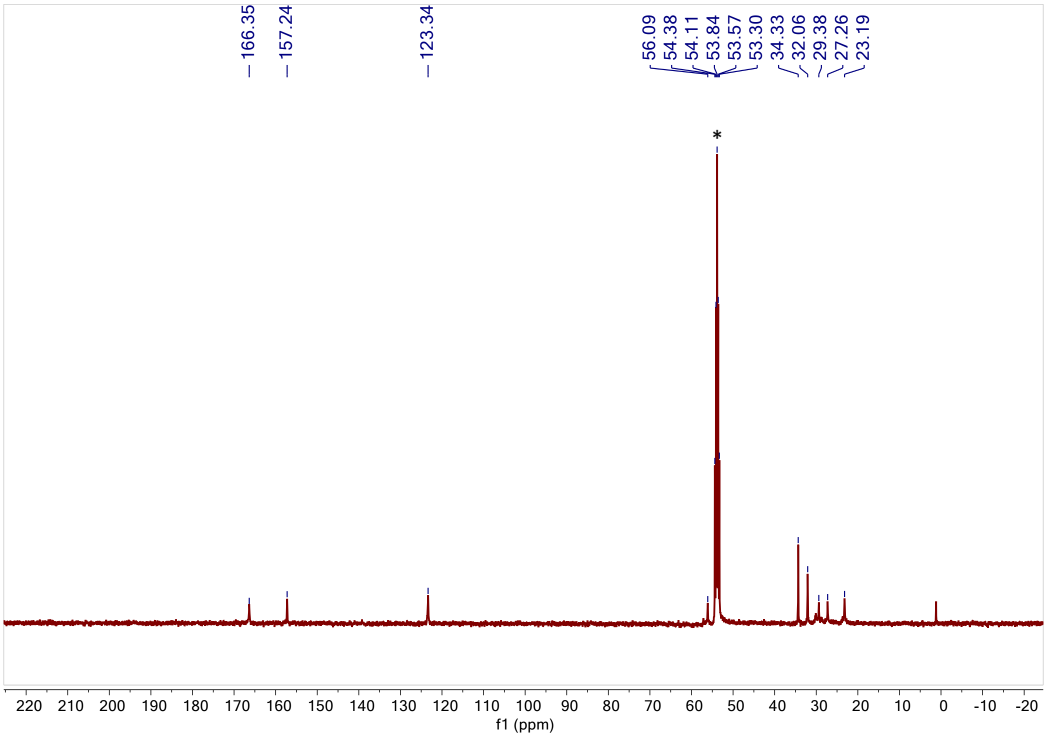

Figure S19. ^{13}C NMR spectra of $[^{6}\mathbf{L}Cu_2]_3$ in CD_2Cl_2 (100 MHz, 298 K). Solvent residual peaks are marked with an asterisk (*).

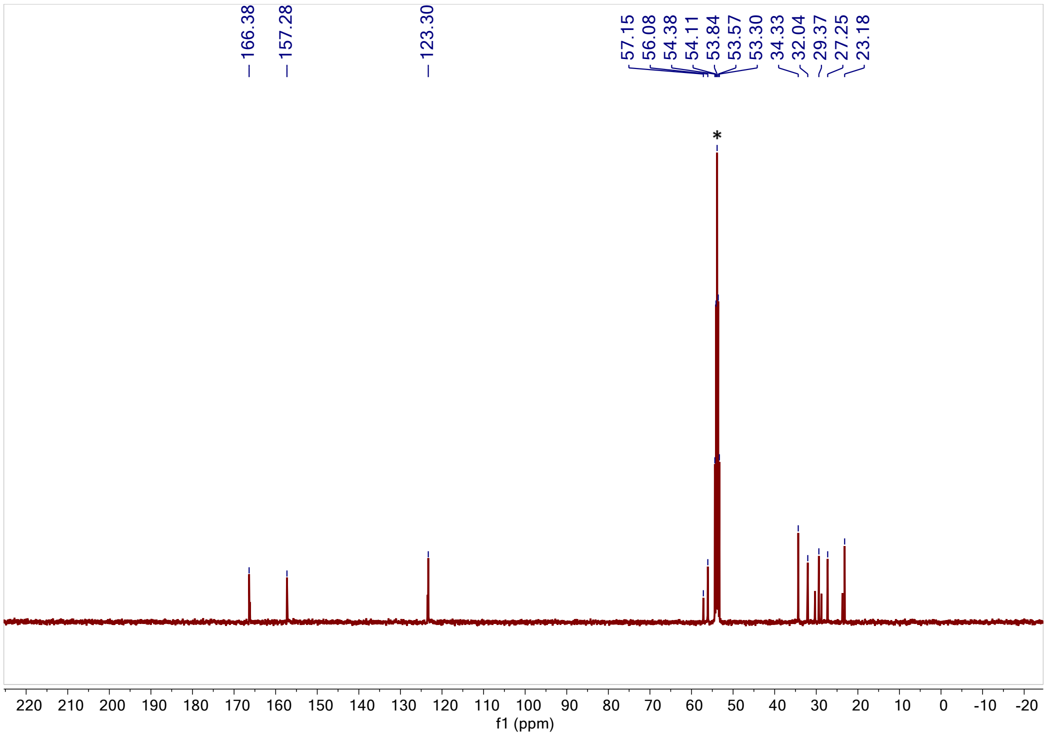

Figure S20. ^{13}C NMR spectra of [^{8}**L**Cu_2]$_3$ in CD_2Cl_2 (100 MHz, 298 K). Solvent residual peaks are marked with an asterisk (*).

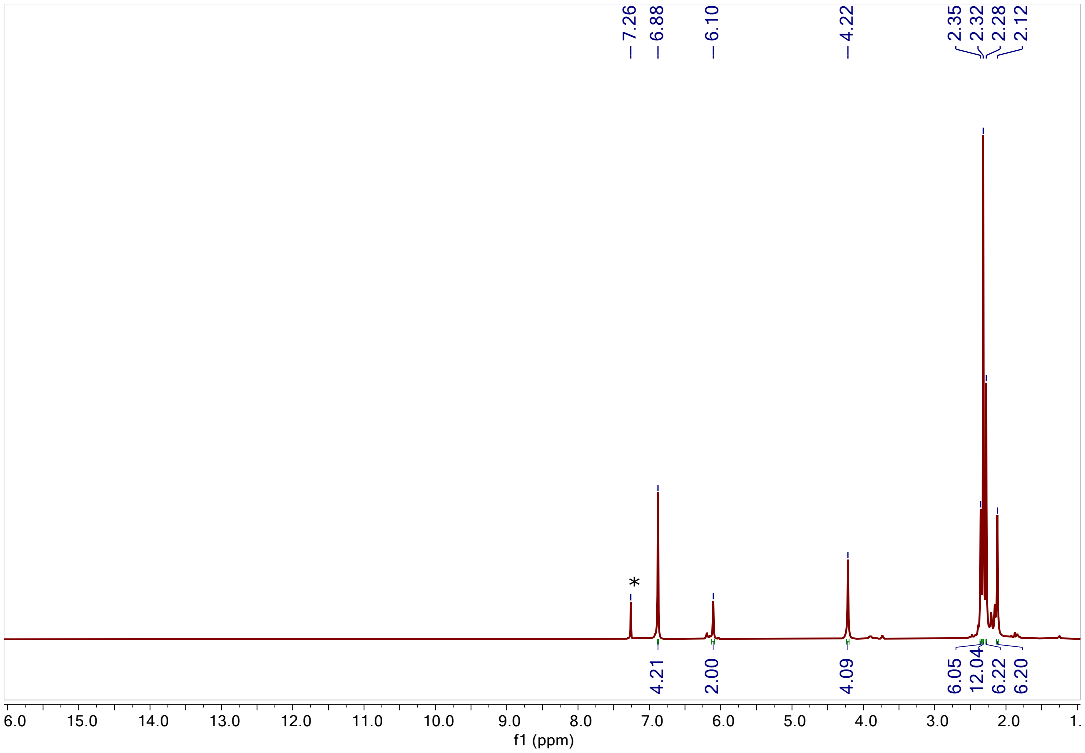

Figure S21. ^{1}H NMR spectra of [^{2}**L**(Cu-CNR)$_2$] in $CDCl_3$ (400 MHz, 298 K). Solvent residual peaks are marked with an asterisk (*).

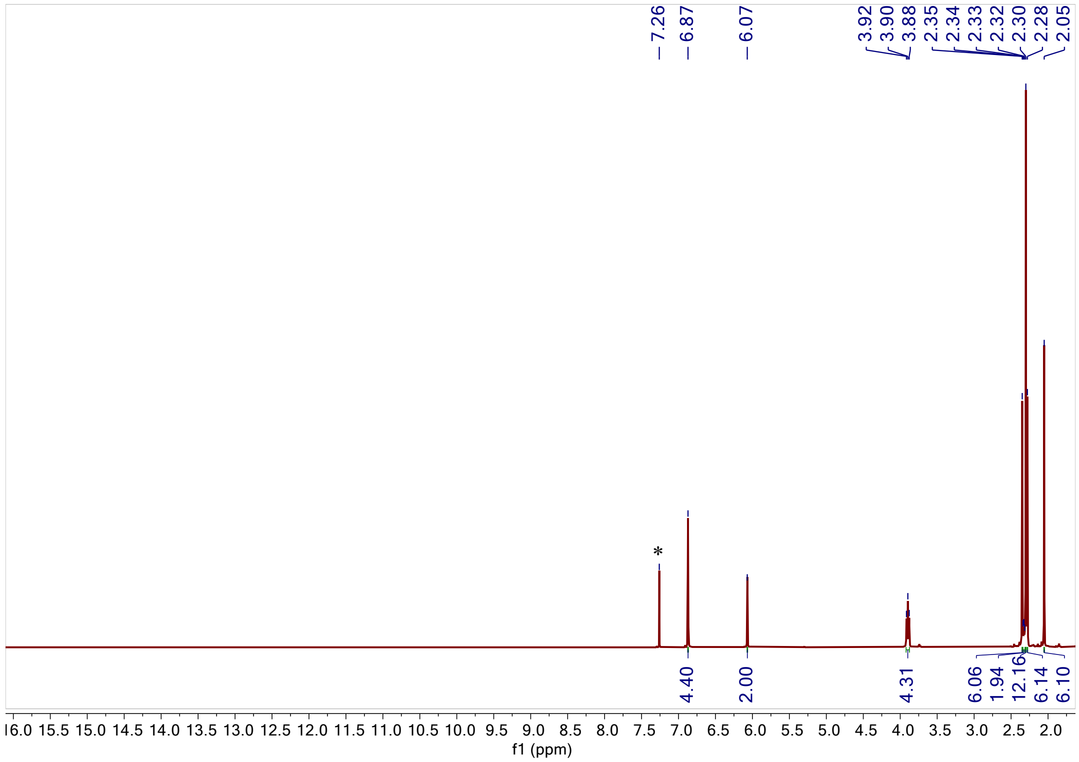

Figure S22. ^{1}H NMR spectra of [^{3}**L**(Cu-CNR)$_2$] in $CDCl_3$ (100 MHz, 298 K). Solvent residual peaks are marked with an asterisk (*).

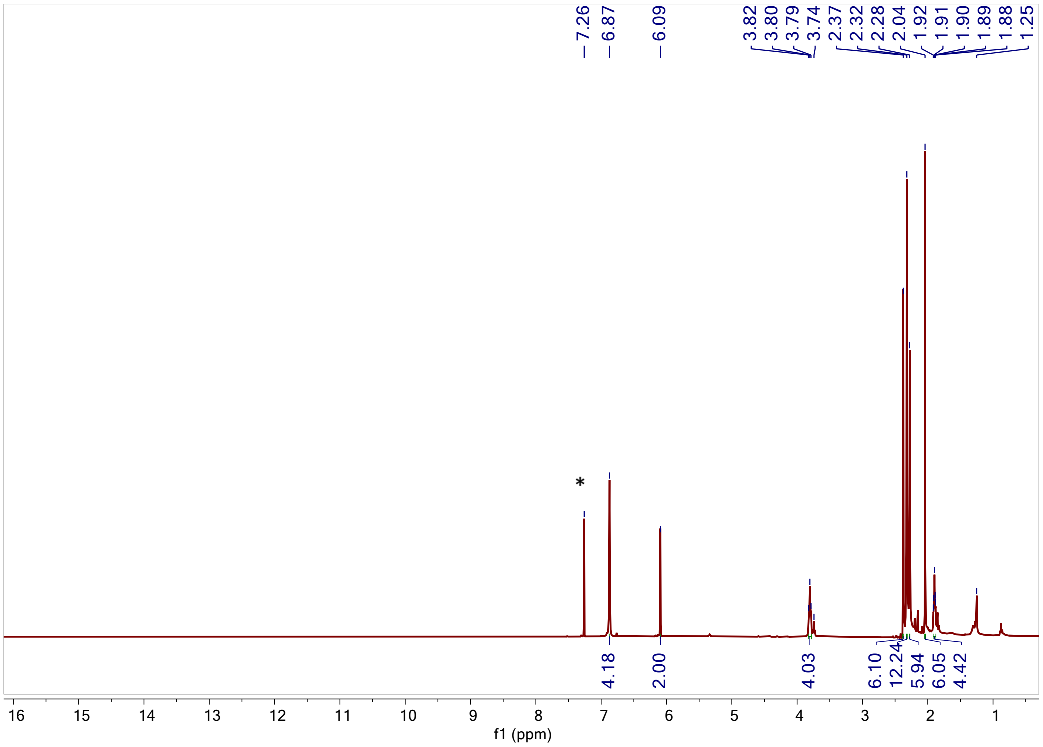

Figure S23. ^{1}H NMR spectra of [^{4}**L**(Cu-CNR)$_2$] in $CDCl_3$ (400 MHz, 298 K). Solvent residual peaks are marked with an asterisk (*).

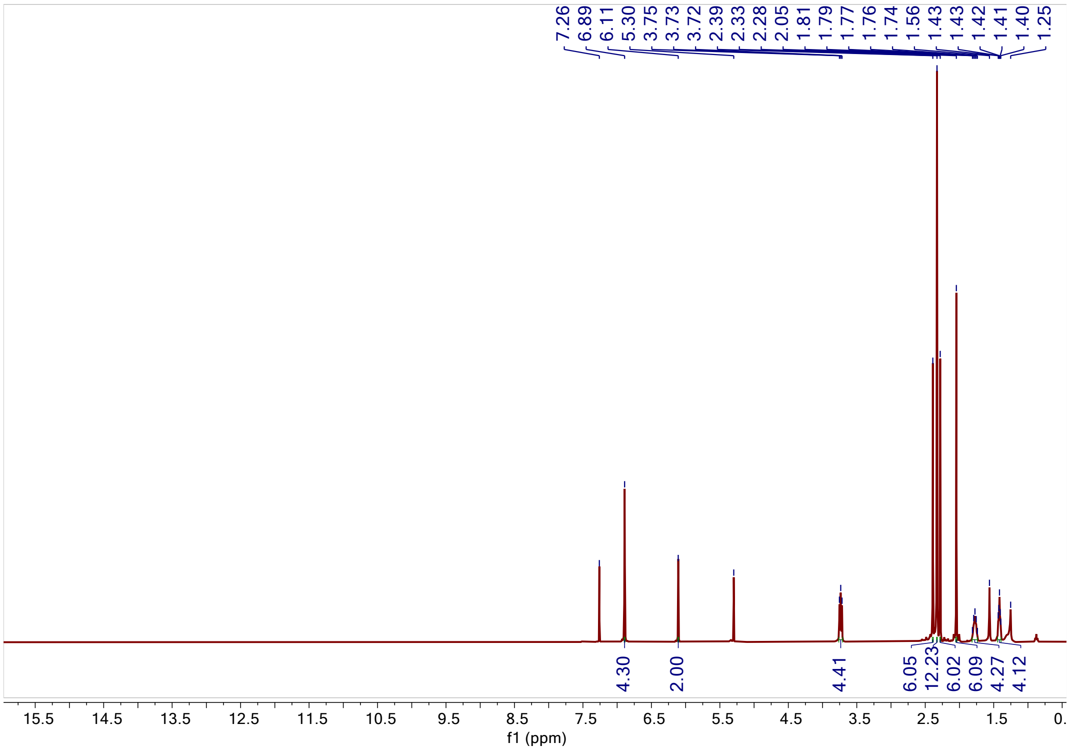

Figure S24. ^{1}H NMR spectra of [^{6}**L**(Cu-CNR)$_2$] in $CDCl_3$ (400 MHz, 298 K). Solvent residual peaks are marked with an asterisk (*).

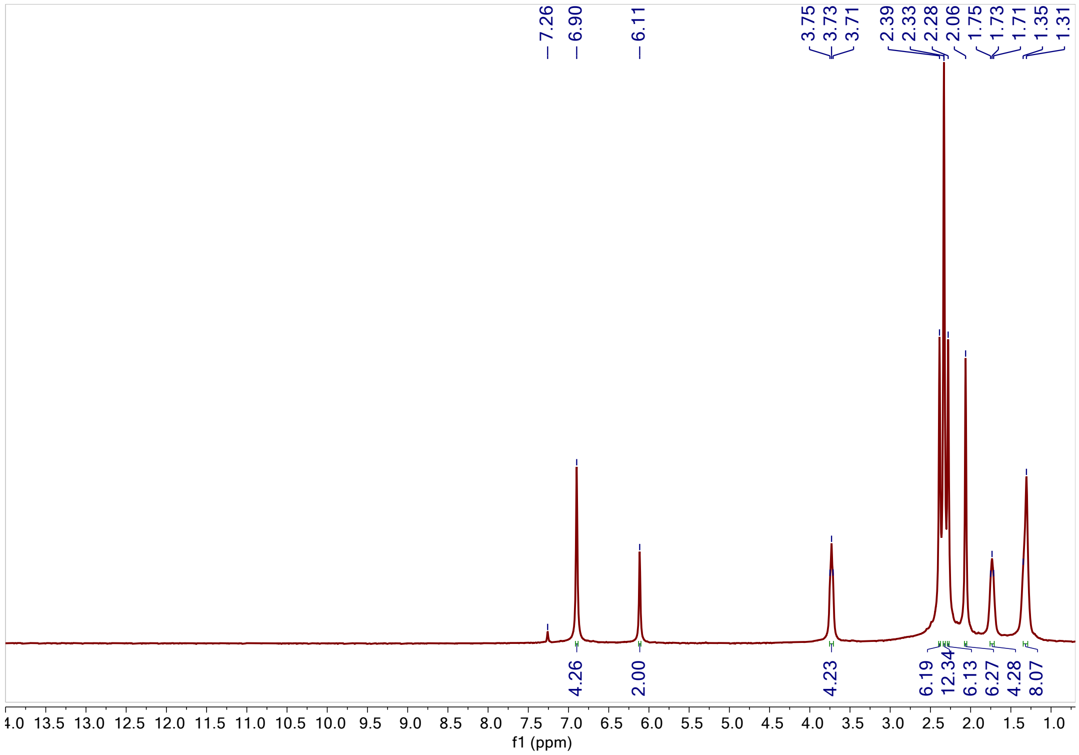

Figure S25. ^{1}H NMR spectra of [^{8}**L**(Cu-CNR)$_2$] in $CDCl_3$ (100 MHz, 298 K). Solvent residual peaks are marked with an asterisk (*).

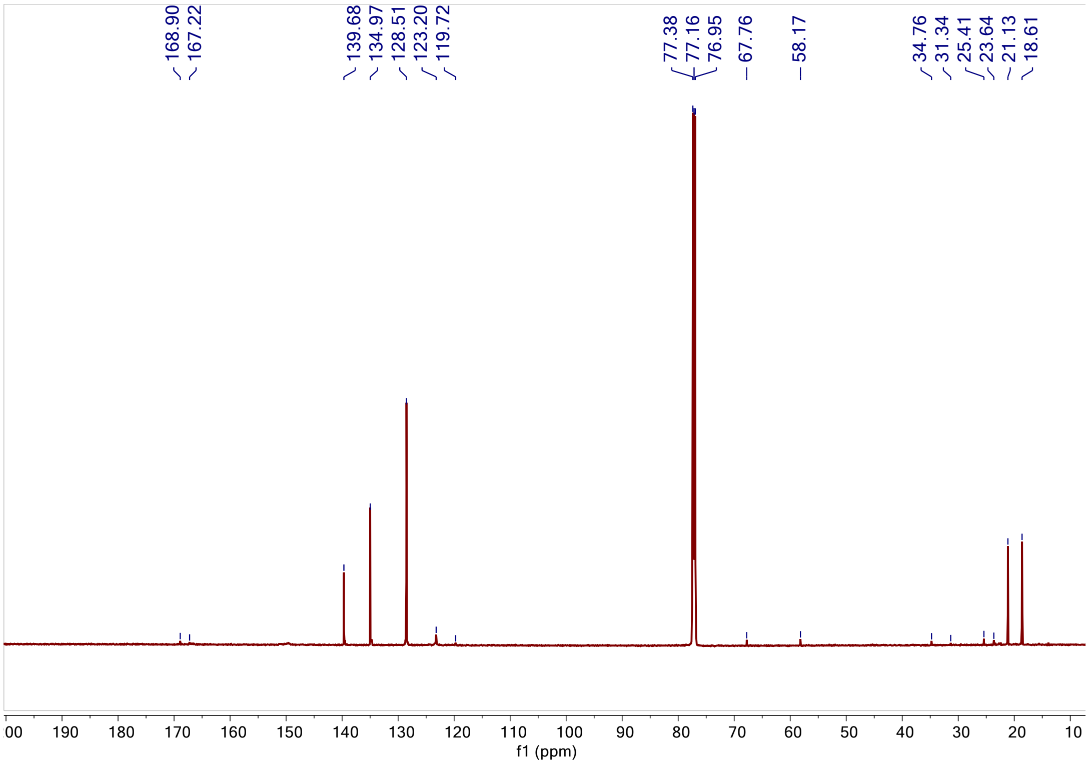

Figure S26. ^{13}C NMR spectra of [^{2}**L**(Cu-CNR)$_2$] in $CDCl_3$ (100 MHz, 298 K). Solvent residual peaks are marked with an asterisk (*).

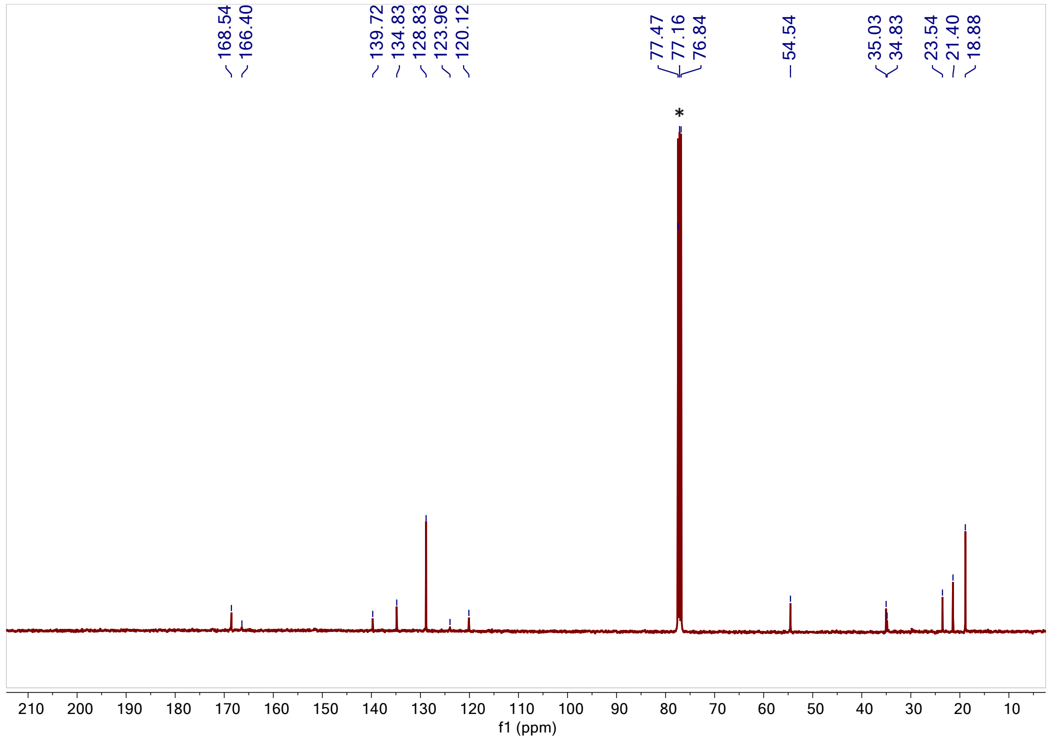

Figure S27. ^{13}C NMR spectra of [^{3}**L**(Cu-CNR)$_2$] in $CDCl_3$ (100 MHz, 298 K). Solvent residual peaks are marked with an asterisk (*).

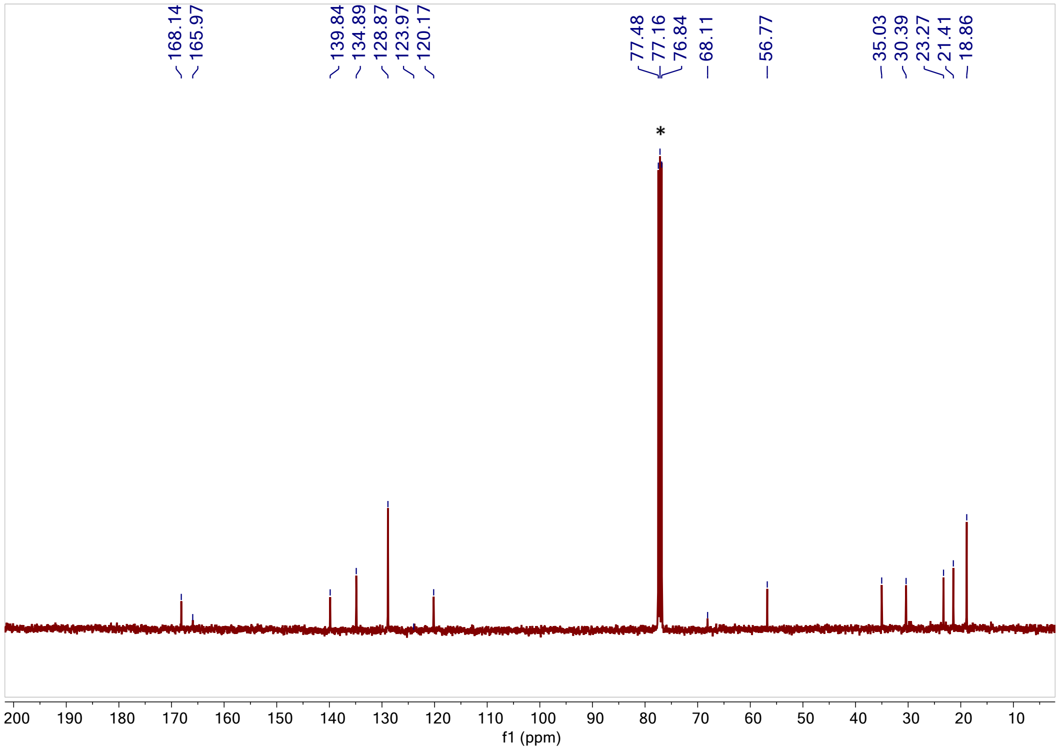

Figure S28. ^{13}C NMR spectra of [^{4}**L**(Cu-CNR)$_2$] in $CDCl_3$ (100 MHz, 298 K). Solvent residual peaks are marked with an asterisk (*).

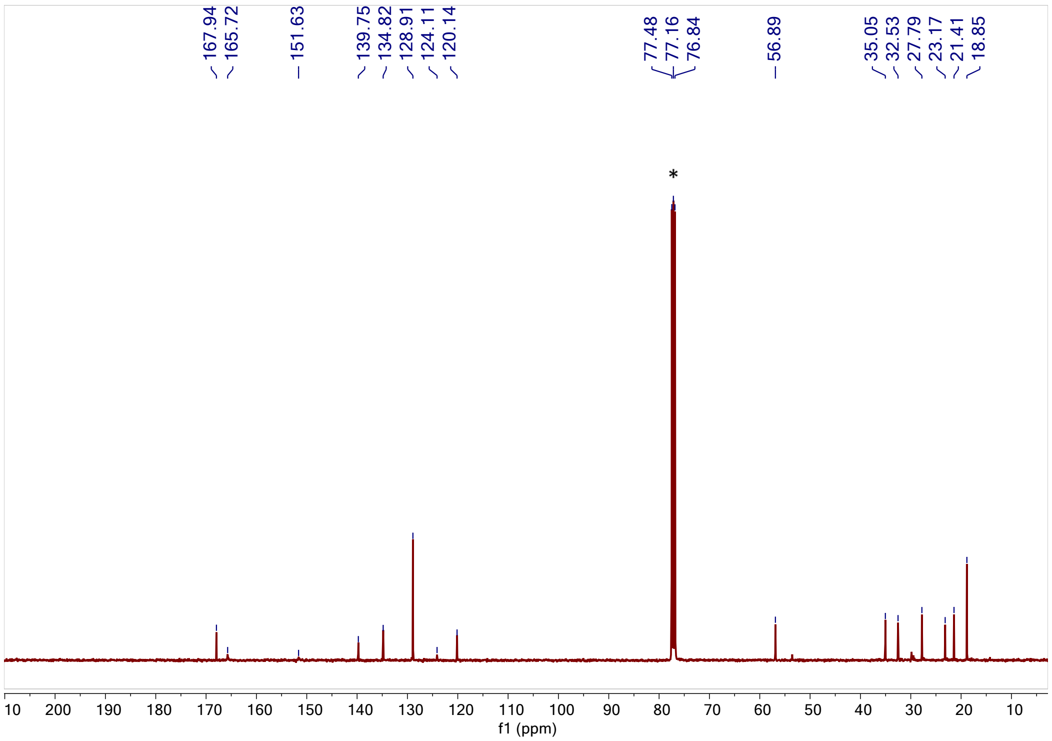

Figure S29. ^{13}C NMR spectra of [^{6}**L**(Cu-CNR)$_2$] in $CDCl_3$ (100 MHz, 298 K). Solvent residual peaks are marked with an asterisk (*).

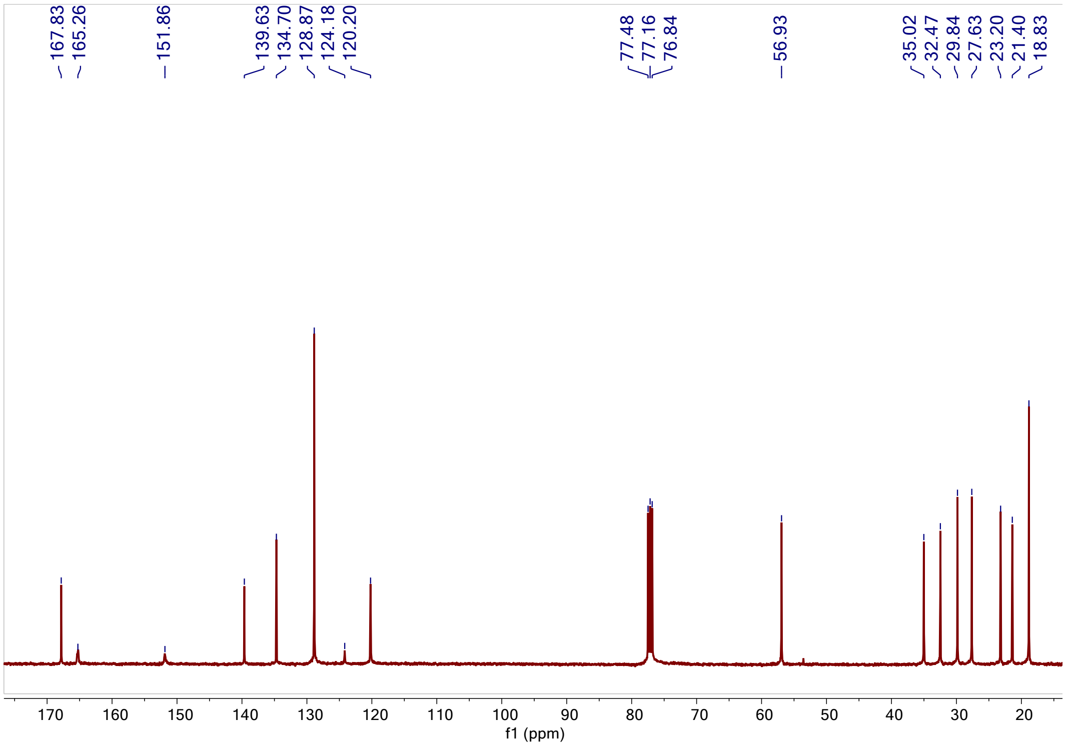

Figure S30. ^{13}C NMR spectra of [^{8}**L**(Cu-CNR)$_2$] in $CDCl_3$ (100 MHz, 298 K). Solvent residual peaks are marked with an asterisk (*).

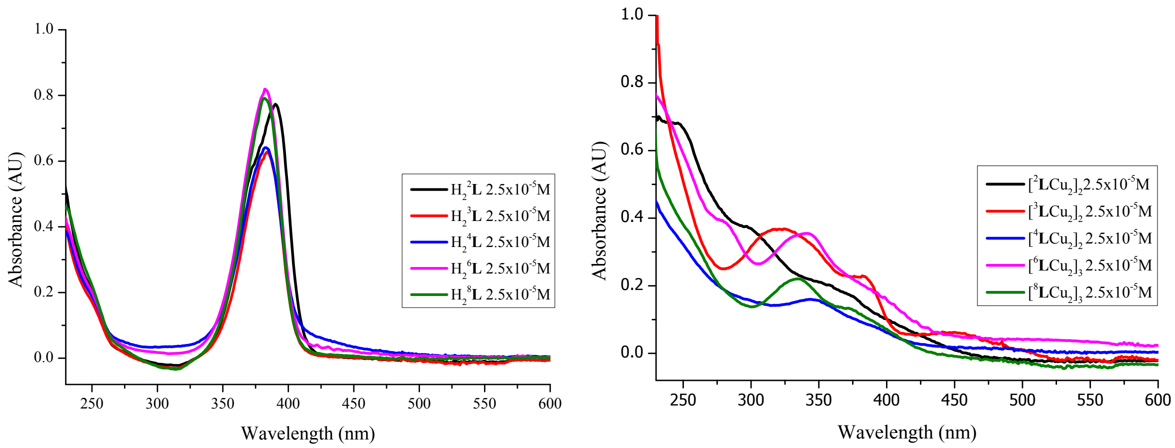

(A) (B)

Figure S31. UV-Vis spectra of the thiolate ligands H_2^{2}**L**-H_2^{4}**L**, H_2^{6}**L** and H_2^{8}**L** (A), while the spectra of the corresponding copper(I) thiolate complexes **1-5** (B).

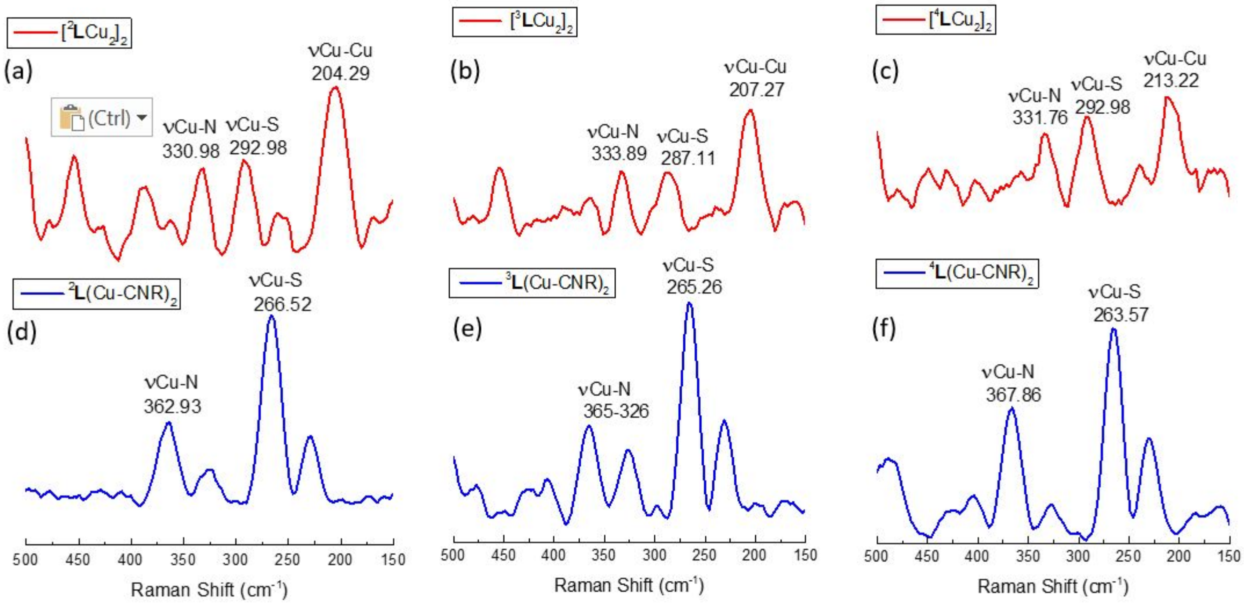

Figure S32. Raman spectra of [**L**Cu_2]$_2$, complexes (red) and their corresponding isocyanide adducts [**L**(Cu-CNR)$_2$] (blue): (a) [^{2}**L**Cu_2]$_2$, (b) [^{3}**L**Cu_2]$_2$, (c) [^{4}**L**Cu_2]$_2$, (d) [^{2}**L**(Cu-CNR)$_2$], (e) [^{3}**L**(Cu-CNR)$_2$], (f) [^{4}**L**(Cu-CNR)$_2$].

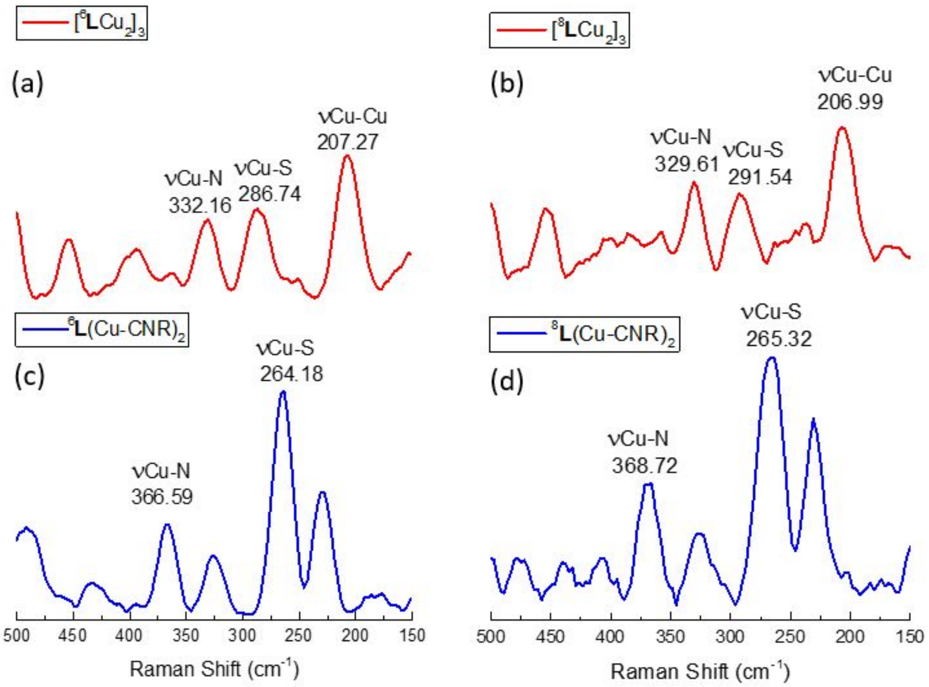

Figure S33. Raman spectra of [$\mathbf{L}Cu_2$]$_3$, complexes (red) and their corresponding isocyanide adducts [$\mathbf{L}$(Cu-CNR)$_2$] (blue): (a) [$^6\mathbf{L}Cu_2$]$_3$, (b) [$^8\mathbf{L}Cu_2$]$_3$, (c) [$^6\mathbf{L}$(Cu-CNR)$_2$], and (d) [$^8\mathbf{L}$(Cu-CNR)$_2$].

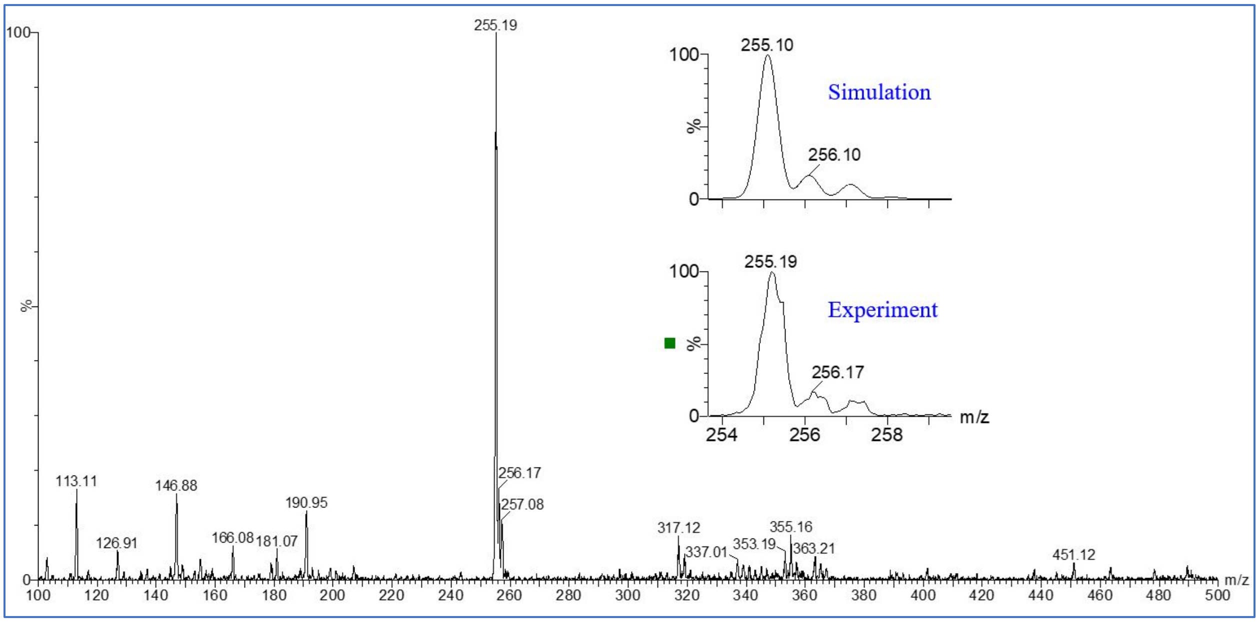

Figure S34. ESI-MS spectra of the thiolate ligand $H_2{}^2\mathbf{L}$. The upper spectrum of the inserted represents the simulated isotopic pattern, while the lower spectrum of the inserted shows the experimental result.

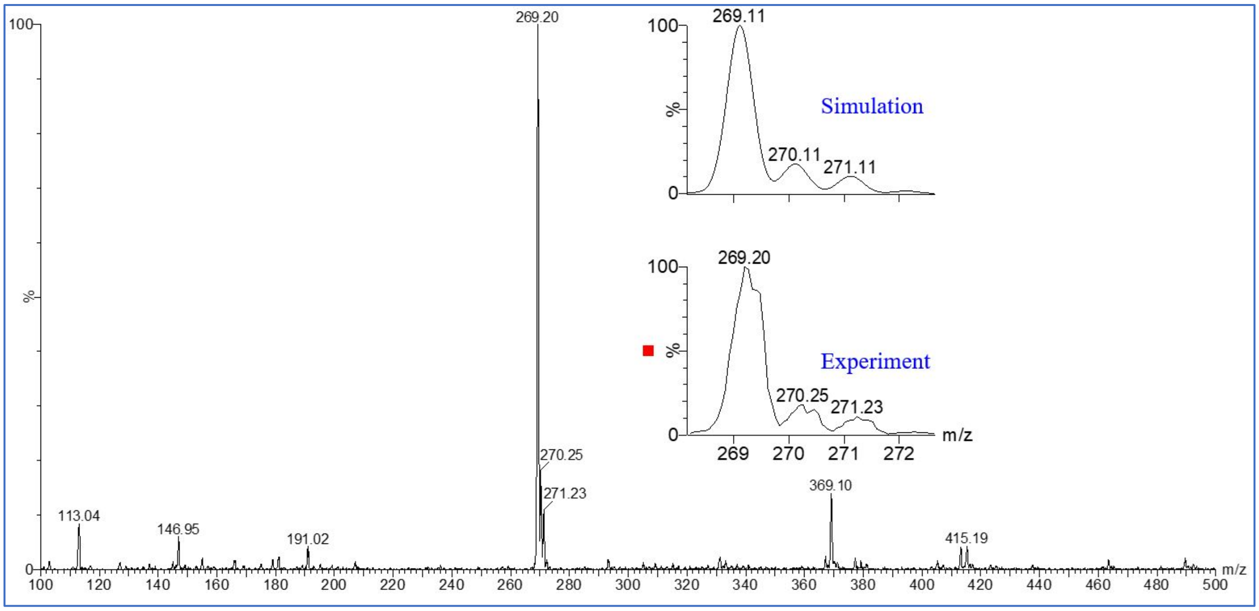

Figure S35. ESI-MS spectra of the thiolate ligand $H_2{}^3$**L**. The upper spectrum of the inserted represents the simulated isotopic pattern, while the lower spectrum of the inserted shows the experimental result.

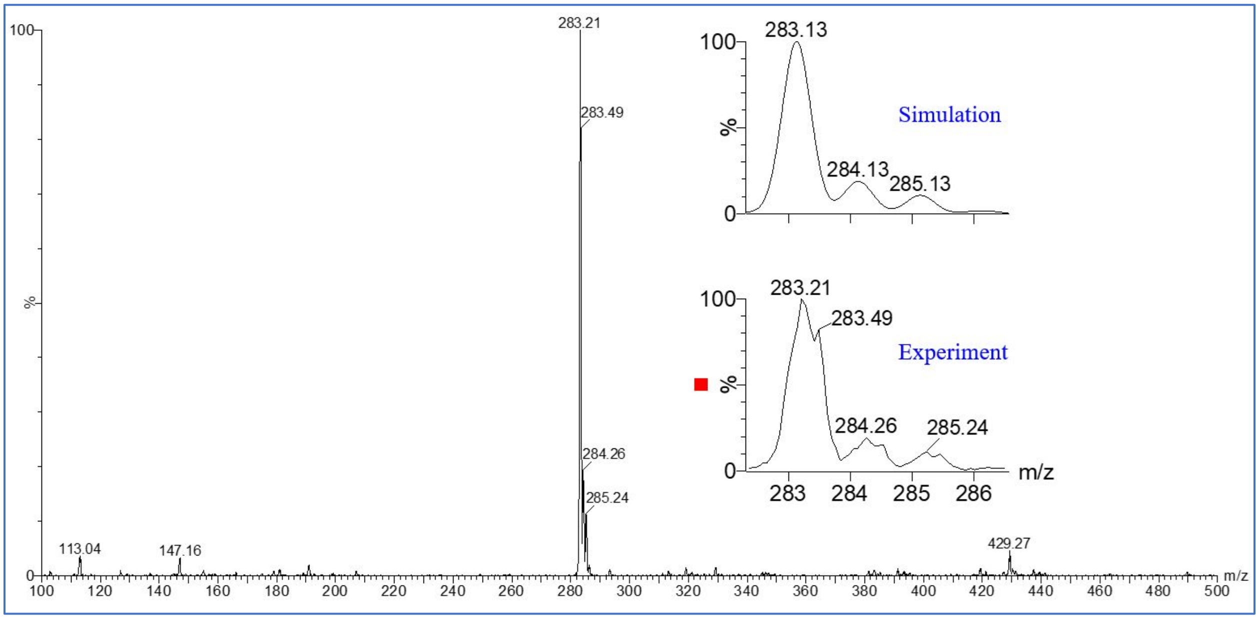

Figure S36. ESI-MS spectra of the thiolate ligand $H_2{}^4$**L**. The upper spectrum of the inserted represents the simulated isotopic pattern, while the lower spectrum of the inserted shows the experimental result.

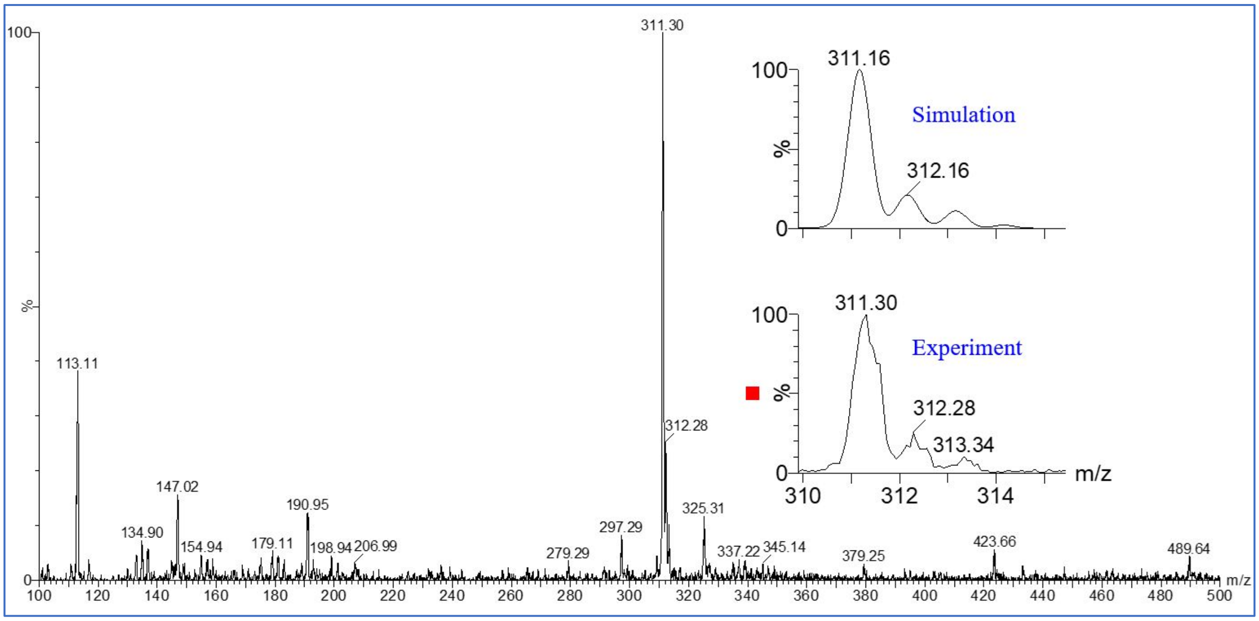

Figure S37. ESI-MS spectra of the thiolate ligand $H_2{}^6\mathbf{L}$. The upper spectrum of the inserted represents the simulated isotopic pattern, while the lower spectrum of the inserted shows the experimental result.

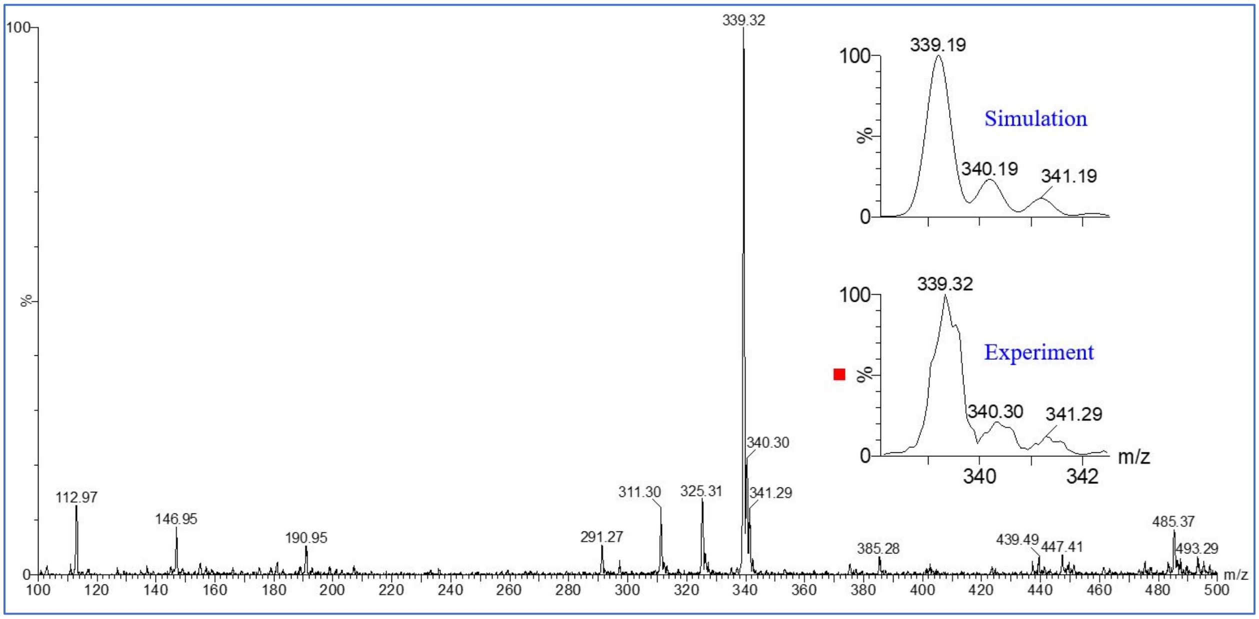

Figure S38. ESI-MS spectra of the thiolate ligand $H_2{}^8\mathbf{L}$. The upper spectrum of the inserted represents the simulated isotopic pattern, while the lower spectrum of the inserted shows the experimental result.

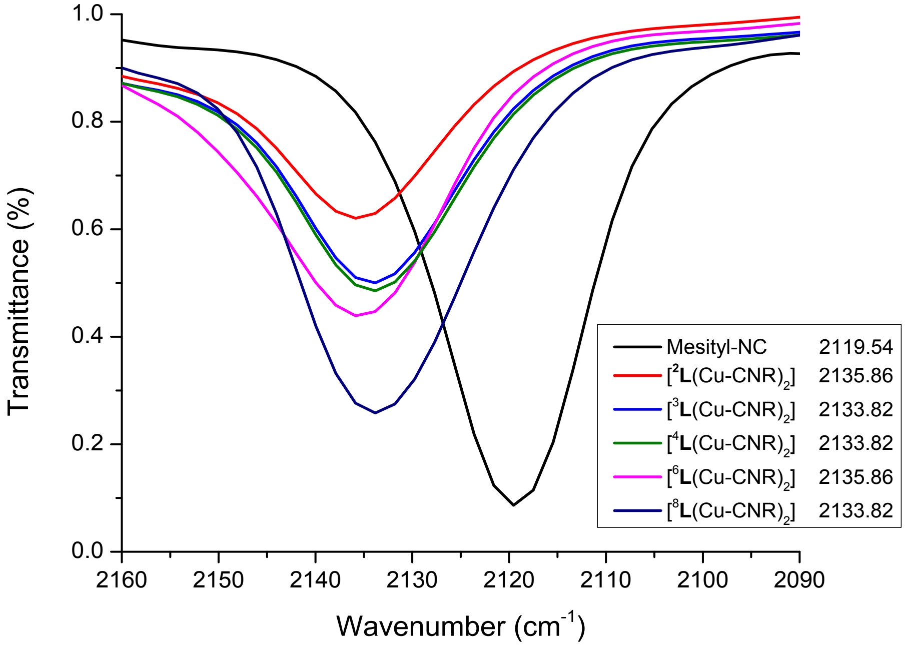

Figure S39. FT-IR spectra of 2,4,6-trimethylphenylisocyanide (Mesityl-NC) and its Copper(I) complexes [^{2}**L**(Cu-CNR)$_2$], [^{3}**L**(Cu-CNR)$_2$], [^{4}**L**(Cu-CNR)$_2$], [^{6}**L**(Cu-CNR)$_2$], and [^{8}**L**(Cu-CNR)$_2$].

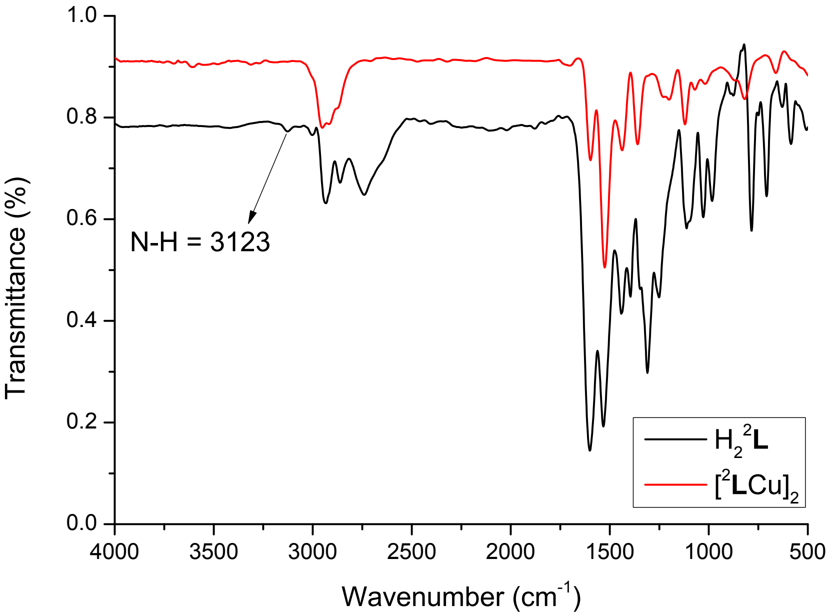

Figure S40. Comparative FT-IR spectra of the uncoordinated ligand $H_2{}^2$**L** (black trace, bottom) and its corresponding copper(I) complex [^{2}**L**Cu_2]$_2$. (red trace, top).

Table S1 Crystal data and structure refinement parameters for the Cu(I) β-thioketiminate complexes.

**Structural parameter**	$[^{2}\mathbf{L}Cu_2]_2$[a]	$[^{4}\mathbf{L}Cu_2]_2$	$[^{6}\mathbf{L}Cu_2]_3$[b]	$[^{8}\mathbf{L}Cu_2]_3$[c]	$^{6}\mathbf{L}(Cu\text{-}CNR)_2$	$^{8}\mathbf{L}(Cu\text{-}CNR)_2$
Empirical formula	$C_{28}H_{44}Cu_4N_4OS_4$	$C_{30}H_{47}Cu_4N_5S_4$	$C_{51}\ H_{69}\ Cu_6\ N_6\ S_6$	$C_{58}\ H_{96}\ Cu_6\ N_8\ S_6$	$C_{18}H_{24}CuN_2S$	$C_{45}H_{60}Cu_2N_4S_2$
Formula weight	835.07	860.12	1339.72	1479.02	363.99	848.17
Temperature/K	130(2)	130(2)	100(2) K	200(2) K	100.00	130(2)
Crystal system	Monoclinic	Monoclinic	Hexagonal	Monoclinic	Triclinic	Monoclinic
Space group	I2/a	$P2_1/c$	$P6_3/m$	C 2/c	P-1	$P2_1/c$
a/Å	30.5619(6)	9.6286(3)	15.1100(6) Å	13.0174(6) Å	8.6204(5)	15.4383(3)
b/Å	10.6561(2)	37.8211(11)	15.1100(6) Å	20.4623(7) Å	8.7166(5)	19.1910(4)
c/Å	22.9473(4)	9.7987(3)	19.5173(12) Å	26.9687(11) Å	12.8278(8)	15.7106(4)
α/°, β/°, γ/°	90, 107.3(2), 90	90, 90.2(3), 90	90°, 90°, 120°	90°, 99.33(10)°, 90°	91.0(2), 93(2), 103(2)	90, 105 (2), 90
Volume/Å^3	7135.3(2)	3568.31(19)	3859.0(4) Å^3	7088.4(5) Å^3	937.20(10)	4495.42(18)
Z	8	4	2	4	2	4
ρ_{calc}g/cm^3	1.555	1.601	1.153	1.386	1.290	1.253
μ/mm^{-1}	2.613	2.614	1.815	1.984	1.275	1.073
F(000)	3424.0	1768.0	1374	3080	382.0	1792.0
Crystal size/mm^3	0.3 × 0.2 × 0.2	0.5 × 0.4 × 0.2	0.130 x 0.100 x 0.057	0.200 x 0.140 x 0.030	0.333 × 0.091 × 0.063	0.5 × 0.3 × 0.2
Radiation	Mo Kα (λ = 0.71073)	Mo Kα (λ = 0.71073)	Mo Kα (λ = 0.71073)	Mo Kα (λ = 0.71073)	Mo Kα (λ = 0.71073)	Mo Kα (λ = 0.71073)
2Θ range for data collection/°	3.93 to 54.23	4.23 to 54.242	1.874 to 28.302°.	2.756 to 25.116°	4.798 to 56.654	3.92 to 54.214
Index ranges	-38 ≤ h ≤ 37, -13 ≤ k ≤ 12, -27 ≤ l ≤ 29	-11 ≤ h ≤ 12, -47 ≤ k ≤ 48, -12 ≤ l ≤ 12	-16<=h<=20, -20<=k<=19, -26<=l<=26	-15<=h<=15, -24<=k<=24, -32<=l<=32	-11 ≤ h ≤ 11, -11 ≤ k ≤ 11, -17 ≤ l ≤ 17	-19 ≤ h ≤ 18, -24 ≤ k ≤ 24, -20 ≤ l ≤ 19
Reflections collected	41516	31947	56160	49581	32905	51373
Independent reflections	7452 [R_{int} = 0.0374, R_{sigma} = 0.0338]	7230 [R_{int} = 0.1042, R_{sigma} = 0.0618]	3292 [R(int) = 0.0528]	6264 [R(int) = 0.0608]	4648 [R_{int} = 0.0376, R_{sigma} = 0.0227]	9448 [R_{int} = 0.0708, R_{sigma} = 0.0603]
Data/restraints/parameters	7452/127/378	7230/0/398	3292 / 13 / 108	6264 / 3 / 349	4648/0/204	9448/0/489
Goodness-of-fit on F^2	1.074	1.058	1.051	1.019	1.048	1.032
Final R indexes [I>=2σ (I)]	R_1 = 0.0313, wR_2 = 0.0735	R_1 = 0.0505, wR_2 = 0.1218	R1 = 0.0996, wR2 = 0.2596	0.0541, wR2 = 0.1539	0.0321, wR_2 = 0.0838	0.0455, wR_2 = 0.0973
Final R indexes [all data]	R_1 = 0.0439, wR_2 = 0.0776	R_1 = 0.0644, wR_2 = 0.1276	R1 = 0.1587, wR2 = 0.3072	0.0789, wR2 = 0.1751	0.0364, wR_2 = 0.0869	0.0958, wR_2 = 0.1113
Largest diff. peak/hole / e Å^{-3}	0.56/-0.31	0.58/-0.78	1.012 and -0.644 e.Å^{-3}	1.028 and -0.432 e.Å^{-3}	0.65/-0.42	0.23/-0.30

[a] **Complex 1 $[^{2}L\cdot Cu_2]_2$.** The B-level alert arises from the short C(sp^3)-C(sp^3) distance (C27-C28) arises from the disordered THF solvent molecule in the lattice. Overlapping disorder components produce an artificially short bond

during refinement. This effect is limited to the solvent region and does not impact the molecular structure of compound **1**.

[b] **Complex 4 [^{6}LCu$_2$]$_3$** The B-level alert arises from low C-C bond precision caused by the long, flexible aliphatic chain in this large multinuclear cluster. Increased thermal motion at the periphery leads to higher uncertainties in C-C distances. All key structural features, including the Cu_6 core, remain well defined and chemically reliable.

[c] **Complex 5 [^{8}LCu$_2$]$_3$** The B-level alert arises from the isotropic refinement of C24A, caused by partial disorder and high thermal motion in the long carbon arm of the ligand. Anisotropic refinement produced unstable parameters, so isotropic treatment was required. This localized issue does not affect the accuracy of the overall structure.

Table S2. Selected bond distances (Å) and bond angles (°) for the ligands [^{2}LCu$_2$]$_2$, [^{4}LCu$_2$]$_2$, [^{6}LCu$_2$]$_3$ and [^{8}LCu$_2$]$_3$.

	[^{2}**L**Cu$_2$]$_2$	[^{4}**L**Cu$_2$]$_2$	[^{6}**L**Cu$_2$]$_3$	[^{8}**L**Cu$_2$]$_3$
Cu-Cu	2.8542(4), 2.5864(4), 2.7338(4), 2.7334 (4), 2.6281 (4), 2.9629(5)	2.7036(8), 2.8023(7), 2.8595 (8), 2.7241 (8), 3.435, 3.368	2.820(2)	2.7865(10), 2.8723(9), 2.7646(9), 2.7647(9), 2.7865(10), 2.8723(9)
Cu-S$_{(chelated)}$	2.2546(7), 2.2434(8), 2.2051 (7), 2.2218(8)	2.1921(13), 2.1864(13), 2.1808(13),2.1866 (13)	2.213(3)	2.1940(15), 2.2026(16), 2.2023(17)
Cu-S$_{(bridged)}$	2.2600(7), 2.2596(7), 2.2704(7) 2.2648(8)	2.2586(13), 2.2731(13), 2.2615 (13), 2.2502 (13)	2.215(3)	2.2277(15), 2.2196(16), 2.2189(15)
Cu-N	1.9794(19), 1.988(2), 1.968(2), 1.975(2)	2.014(4), 2.014(4), 2.011(3), 2.009(4)	1.976(8)	1.961(5), 1.965(4), 1.963(4)
S$_{(chelate)}$CuS$_{(bridge)}$	117.66(3), 120.53(3), 135.40(3), 129.73(3)	138.93(5), 137.15(5), 138.52(5), 136.47(5)	124.30(14)	124.52(6), 124.70(6), 123.03(7)
N-Cu-S$_{(bridge)}$	135.64(6) 132.85(6), 117.15(7), 123.10(7)	112.89(12), 113.33(11), 110.05(11), 113.59(11)	131.0(3)	128.76(14), 128.99(14), 128.10(15)
N-Cu-S$_{(chelate)}$	105.39(6), 105.69(6), 105.46(7), 105.69(7)	104.43(12), 104.64(11), 105.83(11), 105.13(11)	103.9(3)	105.39(14), 105.56(14) 106.16(15)

Table S3. Selected bond distances (Å) and bond angles (°) for complexes [^{n}**L**(Cu-CNR)$_2$ adducts

	[^{6}**L**(Cu-CNR)$_2$	[^{8}**L**(Cu-CNR)$_2$
Cu−N(amide(alkyl))	1.957(14)	2.1899(8), 2.1951(8)
Cu−S	2.203(5)	1.961(2), 1.959(2)
C−C(NCCCS backbone)	1.443(2)<br>1.363(3)	1.439(4), 1.431(4)<br>1.345(4), 1.345(4)
C−N	1.311(2)	1.318(3), 1.315(3)
C−S	1.715(2)	1.720(3), 1.728(3)
Cu−C(isocyanide)	1.850(19)	1.849(3), 1.845(3)
C≡N(isocyanide)	1.159(2)	1.146(3), 1.154(3)
N(amide(aryl))−Cu−S	105.27(4)	105.77(6), 105.70(7)
C(isocyanide)−Cu−S	135.77(7)	120.76(9), 119.67(9)
C(isocyanide)−Cu−N(amide(alkyl))	118.93(6)	133.44(10), 134.58(11)
C(amide)-N-C(aryl)	115.75(14)	116.8(2), 116.6(2)
∠NCCCS(mesityl)[a]	3.66	3.78, 10.13

[c]Dihedral angle between NCCCS plane and the mesityl ring
